# The Intronic Long Noncoding RNA *ANRASSF1* Recruits PRC2 to the *RASSF1A* Promoter, Reducing the Expression of *RASSF1A* and Increasing Cell Proliferation

**DOI:** 10.1371/journal.pgen.1003705

**Published:** 2013-08-22

**Authors:** Felipe C. Beckedorff, Ana C. Ayupe, Renan Crocci-Souza, Murilo S. Amaral, Helder I. Nakaya, Daniela T. Soltys, Carlos F. M. Menck, Eduardo M. Reis, Sergio Verjovski-Almeida

**Affiliations:** 1 Departamento de Bioquímica, Instituto de Química, Universidade de São Paulo, São Paulo, São Paulo, Brasil; 2 Departamento de Microbiologia, Instituto de Ciências Biomédicas, Universidade de São Paulo, São Paulo, São Paulo, Brasil; 3 Instituto Nacional de Ciência e Tecnologia em Oncogenômica, São Paulo, São Paulo, Brasil; Massachusetts General Hospital, Howard Hughes Medical Institute, United States of America

## Abstract

The down-regulation of the tumor-suppressor gene *RASSF1A* has been shown to increase cell proliferation in several tumors. *RASSF1A* expression is regulated through epigenetic events involving the polycomb repressive complex 2 (PRC2); however, the molecular mechanisms modulating the recruitment of this epigenetic modifier to the *RASSF1* locus remain largely unknown. Here, we identify and characterize *ANRASSF1*, an endogenous unspliced long noncoding RNA (lncRNA) that is transcribed from the opposite strand on the *RASSF1* gene locus in several cell lines and tissues and binds PRC2. *ANRASSF1* is transcribed through RNA polymerase II and is 5′-capped and polyadenylated; it exhibits nuclear localization and has a shorter half-life compared with other lncRNAs that bind PRC2. *ANRASSF1* endogenous expression is higher in breast and prostate tumor cell lines compared with non-tumor, and an opposite pattern is observed for *RASSF1A*. *ANRASSF1* ectopic overexpression reduces *RASSF1A* abundance and increases the proliferation of HeLa cells, whereas *ANRASSF1* silencing causes the opposite effects. These changes in *ANRASSF1* levels do not affect the *RASSF1C* isoform abundance. *ANRASSF1* overexpression causes a marked increase in both PRC2 occupancy and histone H3K27me3 repressive marks, specifically at the *RASSF1A* promoter region. No effect of *ANRASSF1* overexpression was detected on PRC2 occupancy and histone H3K27me3 at the promoter regions of *RASSF1C* and the four other neighboring genes, including two well-characterized tumor suppressor genes. Additionally, we demonstrated that *ANRASSF1* forms an RNA/DNA hybrid and recruits PRC2 to the *RASSF1A* promoter. Together, these results demonstrate a novel mechanism of epigenetic repression of the *RASSF1A* tumor suppressor gene involving antisense unspliced lncRNA, in which *ANRASSF1* selectively represses the expression of the *RASSF1* isoform overlapping the antisense transcript in a location-specific manner. In a broader perspective, our findings suggest that other non-characterized unspliced intronic lncRNAs transcribed in the human genome might contribute to a location-specific epigenetic modulation of genes.

## Author Summary

*RASSF1A* is a tumor suppressor gene whose expression is repressed through epigenetic events in a wide range of different cancers. Repression is effected by DNA hypermethylation of the *RASSF1A* promoter, which in turn is triggered through histone H3K9/H3K27 trimethylation repressive marks. The addition of the H3K27me3 mark at the *RASSF1A* promoter locus involves the polycomb repressive complex 2 (PRC2). The molecular mechanisms that control the recruitment of PRC2 to the promoter to initiate H3K27 trimethylation and repress *RASSF1A* expression have not been described. Here, we identified a long noncoding RNA (lncRNA), termed *ANRASSF1* for *antisense noncoding RASSF1*, that is transcribed from the opposite strand of the *RASSF1A* gene and is responsible for recruiting PRC2 to the *RASSF1A* promoter region in a highly location-specific manner. No effect of *ANRASSF1* was detected on the promoter of the *RASSF1C* isoform or the promoters of the four other genes within the vicinity of *RASSF1*, including two other well-characterized tumor suppressor genes. This work provides evidence that the epigenetic modulation of the tumor suppressor gene *RASSF1A* is dependent on the lncRNA *ANRASSF1* and highlights the importance of further studies on the involvement of *ANRASSF1* in tumorigenesis.

## Introduction

*RASSF1A* (RAS-association domain family member 1A) is a tumor suppressor gene that modulates a broad range of cellular functions essential for normal growth, such as the maintenance of genomic stability, cell cycle control, the modulation of apoptosis, and cell motility and invasion [1]. *RASSF1A* is one of seven alternatively spliced isoforms (*RASSF1A* to *G*) generated at the gene locus through the differential usage of two promoters or alternative splicing [2], [3]. The biological relevance of only two isoforms, *RASSF1A* and *RASSF1C*, has been demonstrated. Both isoforms are ubiquitously expressed in non-tumor tissues, whereas in tumors and tumor cell lines, the expression of *RASSF1A* is frequently low, leading to increased cell proliferation [3]. *RASSF1A* promoter CpG island hypermethylation and reduced gene expression are frequently observed in a wide range of cancers [4]–[9]. The epigenetic silencing of *RASSF1A* requires the HOXB3-mediated induction of *DNMT3B* DNA methyltransferase expression and the recruitment of the DNMT3B protein to the *RASSF1A* promoter [10]. The recruitment of DNMT3B and the polycomb repressor complex 2 (PRC2) is dependent on MYC proto-oncogene protein, which is bound to the *RASSF1A* promoter [10]. Although MYC is required for PRC2 recruitment to the *RASSF1A* promoter [10], MYC is generally not sufficient to recruit PRC2 [10], [11], and other regulatory factors and mechanisms underlying the recruitment of this epigenetic silencing machinery have not yet been identified.

The human genome encodes thousands of long (>200 nt) noncoding RNAs (lncRNAs) [12], [13] that might function via diverse mechanisms [14]–[16]. Intergenic lncRNAs have been associated with gene silencing through guiding enzymes involved in chromatin remodeling, particularly PRC2, causing the posttranslational modification of histones in target genes [17]–[19]. In addition, recent reports have shown that thousands of lncRNAs are associated with PRC2 and that many of these are sense and antisense intronic lncRNAs [20], [21], not intergenic.

In the present study, we identified a novel unspliced antisense intronic lncRNA, *ANRASSF1*, which is expressed in the *RASSF1* gene locus independently from the protein-coding gene. The modulation of *ANRASSF1* abundance through ectopic overexpression or transient knockdown affected the expression level of *RASSF1A*, with no effect on *RASSF1C*. We demonstrated that *ANRASSF1* formed an lncRNA/DNA hybrid, which mediated the recruitment of SUZ12, a member of PRC2, to the *RASSF1A* promoter. The recruitment of SUZ12 resulted in a marked increase in the H3K27me3 levels only at the *RASSF1A* promoter region, without accumulation of the repressive mark either at the *RASSF1C* promoter or the four neighboring loci. *ANRASSF1*-mediated gene repression occurred in a highly location-specific manner, as only the *RASSF1A* isoform, which overlaps the antisense transcript, was affected.

## Results

### *ANRASSF1* is transcribed antisense to the *RASSF1A* gene

**Figure 1:**
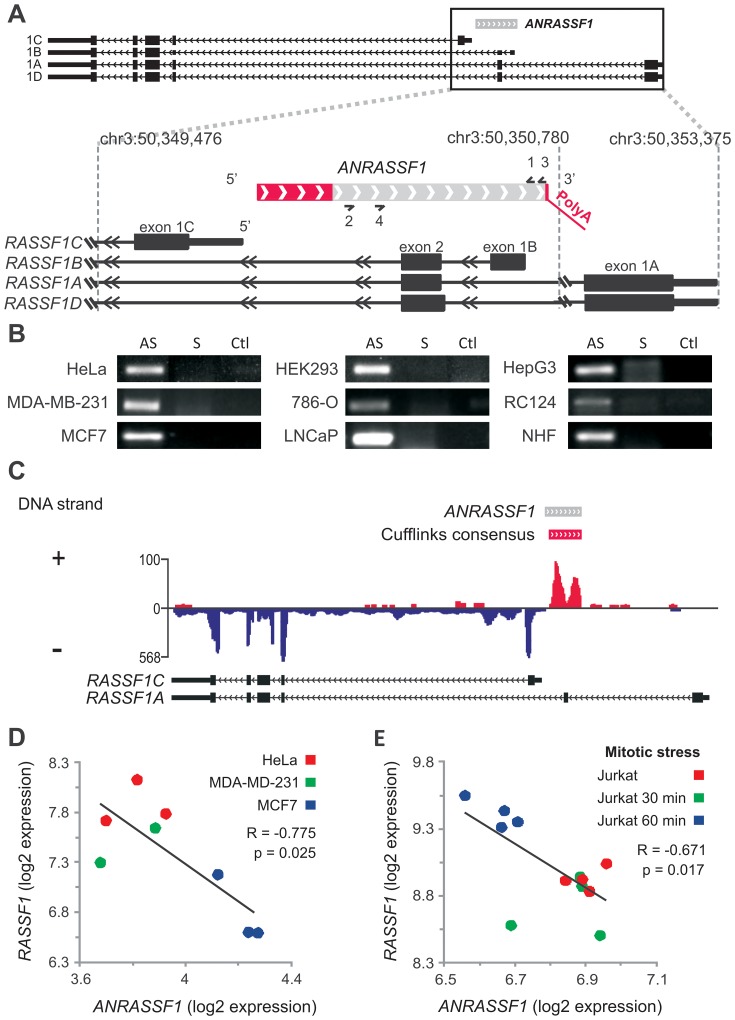
Antisense noncoding RNA *ANRASSF1* is expressed within the *RASSF1* genomic locus and inversely correlated with *RASSF1* expression. (A) Schematic representation of the entire *RASSF1 locus* presented at the top. Protein-coding *RASSF1* gene isoforms (RefSeq-annotated) are shown in black; *ANRASSF1* evidence from an assembly of public ESTs is shown in light gray; the portions extended through 5′-end RACE and primer-walking or 3′-end RACE are shown in red, with a poly(A) segment detected through sequencing. The arrowheads define the orientation of the sequences. (B) *ANRASSF1* was detected in several human cell lines using end-point PCR in the antisense (AS), and not the sense (S), direction relative to the protein-coding gene. Strand-specific primers were used for the reverse-transcription (RT) (short half-arrow numbers 2 and 3 on *ANRASSF1* in panel A). RT reaction in the absence of primer was used as a negative control (Ctl). The short half-arrows numbered 1 and 4 indicate the primers used for the end-point PCR. (C) Strand-specific RNA-seq from poly(A)+ RNA of LNCaP cells. The abundance of the reads mapping within the *RASSF1* genomic locus is displayed in the diagram, and the color corresponds to the DNA strand of the transcripts. The red horizontal bar represents the consensus sequence from the assembly of RNA-seq reads mapping to the positive strand in this locus, generated using the Cufflinks tool. The gray bar represents the full-length *ANRASSF1*, obtained by RACE-PCR and Sanger sequencing. The arrowheads define the orientation of the sequences. (D) Expression of *RASSF1* and *ANRASSF1* measured with Affymetrix microarrays in HeLa, MDA-MB-231 and MCF-7 cells from GSE5823 [54]. (E) The expression of *RASSF1* and *ANRASSF1* measured with Affymetrix microarrays in Jurkat cells under mitotic stress induced through treatment with phorbol ester and ionomycin for 30 and 60 min from GSE11118 [55].

We surveyed the public Expressed Sequence Tags (ESTs) database and identified a long RNA transcript that mapped to an intronic region of the *RASSF1* genomic locus. This transcript was represented by a cluster of ESTs covering 580 bp of the genome, which mapped just upstream of the *RASSF1C* isoform (Figure 1A, light gray rectangle) and overlapped exon 2 of *RASSF1A*. Indeed, this transcript was one of 67,731 putative unspliced lncRNAs mapping to intronic regions of 74% of all protein-coding genes, which were previously described by our group [22], being part of a genome-wide pervasive lncRNA expression involving between 75 and 90% of the human genome [12], [13], [23].

The ESTs described above originated from non-strand specific cDNA libraries produced from a number of different human tissues, and we used strand-oriented RT-PCR to confirm the expression of this long RNA in several cell lines. Figure 1B shows the expression of an RNA transcribed in the antisense direction relative to protein-coding mRNAs encoded in the *RASSF1* locus in nine different cell lines. This transcript will hereafter be referred to as *ANRASSF1*, for *Antisense Intronic Noncoding RASSF1* RNA.

Next, we used the Rapid Amplification of cDNA Ends (RACE) approach to extend both the 5′ and 3′ ends of the *ANRASSF1* transcript. The 3′ RACE-PCR product was sequenced, showing a 38-nt extension beyond the existing ESTs, with 5 nt of the extended sequence matching the genome and the additional sequence showing a poly(A) tail of 33 adenines (Figure 1A). We also identified a conserved polyadenylation signal (ATTAAA) [24] 17 nt upstream of the poly(A) tail. Using a combined approach involving 5′ RACE-PCR with primer-walking PCR and sequencing, we extended the transcript 205 nt at the 5′ end (Figure 1A, red box), resulting in a full-length *ANRASSF1* transcript of 790 nt. High-throughput strand-specific RNA-seq of poly(A)+ RNA from LNCaP prostate cancer cells showed transcription from the plus strand in the *RASSF1* locus (Figure 1C), and the assembly of these RNA-seq reads using the Cufflinks tool generated a consensus sequence mapping to the genomic plus strand in the locus. These data essentially confirmed the length and antisense orientation of *ANRASSF1*, which were previously identified through strand-specific RT-PCR, RACE-PCR and sequencing. Using the Coding Potential Calculator tool [25], no coding potential was predicted for the full-length *ANRASSF1*, confirming *ANRASSF1* as an lncRNA.

Because *ANRASSF1* is represented by an Affymetrix probe set in the HG-U133 Plus2 microarray platform and the entire *RASSF1* locus is represented by a different probe set, we performed a meta-analysis of the *ANRASSF1* and *RASSF1* expression patterns on publicly available microarray data. We identified a statistically significant inverse correlation (Figure 1D) between the expression levels of *ANRASSF1* and *RASSF1* in HeLa, MDA-MB-231 and MCF-7 cells, which are three cell lines in which we had previously confirmed *ANRASSF1* expression using RT-PCR (Figure 1B). In addition, this meta-analysis revealed that in Jurkat cells under mitotic stress (Figure 1E), *RASSF1* showed a 1.5-fold increase within the first hour following mitogen stimulation with a phorbol ester and ionomycin; this response was inversely correlated with *ANRASSF1* expression. Notably, the expression of RASSF1 and Daxx was described to define a mitotic stress checkpoint that enables cells to exit mitosis and eventually die [26]. Our meta-analysis also detected an inverse correlation between *ANRASSF1* and *RASSF1* in three other studies using cell lines and human tissue samples (Figure S1). Overall, these data highlight a functional role for *ANRASSF1* in the host locus.

### Inverse correlation between *ANRASSF1* and *RASSF1A* expression in non-tumor and tumor cell lines

**Figure 2:**
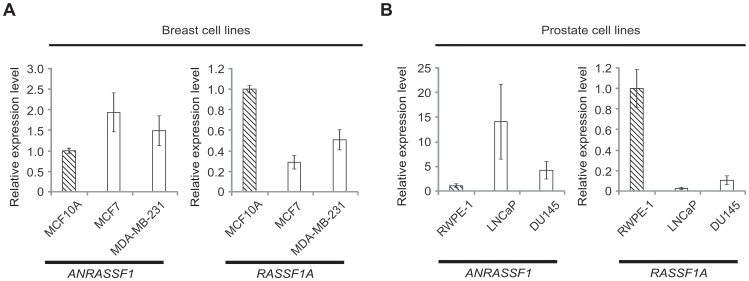
Inverse correlation between *ANRASSF1* and *RASSF1A* expression in non-tumor and tumor cell lines. Expression of *ANRASSF1* and of *RASSF1A* in (A) breast and (B) prostate cell lines. Tumor cell lines (white bars) and non-tumor immortalized cell lines (hatched bars) were tested. These data show the means ± SD from two or three independent biological replicates for each cell line. The expression values in tumors are shown compared with the expression in the non-tumor cell line. These data are calculated relative to *HPRT1* expression.

Next, we measured *ANRASSF1* and *RASSF1A* expression in tumor and non-tumor immortalized cell lines obtained from the breast (Figure 2A) and prostate (Figure 2B). Interestingly, we detected the reduced expression of *ANRASSF1* in non-tumor cell lines compared with tumors in both tissues, and an opposite pattern for *RASSF1A* expression, which was higher in non-tumor cells compared with tumor cell lines. Thus, the inverse correlation between *ANRASSF1* and *RASSF1* expression in the public array datasets was confirmed in the one non-tumor and two tumor cell lines obtained from two different tissues.

### *ANRASSF1* lncRNA is RNAPII-encoded and nuclear-enriched and has a short half-life

To determine whether *ANRASSF1* lncRNA is transcribed through RNA Polymerase II, we treated HeLa cells with α-amanitin at a concentration of 10 µg/mL, which inhibits only RNAPII. Figure S2A shows that *ANRASSF1* transcription was abolished in α-amanitin-treated cells.

*ANRASSF1* contained a 5′ end methyl-guanosine cap modification, as shown by its resistance to 5′-exonucleolytic digestion *in vitro* (Figure S2B). To determine *ANRASSF1* stability, HeLa cell cultures were treated for 1 to 8 h with the RNA polymerase inhibitor actinomycin D. *ANRASSF1* levels decayed with a half-life of ∼50 min following transcriptional inhibition (Figure S2C). For comparison, c-Myc mRNA displayed a half-life of ∼20 min under similar conditions (Figure S2C).

Nuclear and cytoplasmic total RNA fractions were prepared from HeLa cells, and the relative abundance of *ANRASSF1* was measured using qPCR. Figure S2D shows that *ANRASSF1* was 100-fold enriched in the nuclear fraction relative to the cytoplasm.

A comparison of the expression levels of *ANRASSF1* with those of abundant intergenic lncRNAs (lincRNAs), such as *MALAT1* [27], *HOTAIR* [19] and lincRNA *SFPQ* [18] showed that endogenous *ANRASSF1* was much less abundant (approximately 500- to 1,000-fold lower) than these lincRNAs in HeLa cells (Figure S2E). Similarly, endogenous *ANRASSF1* was approximately 500- to 1,000-fold less abundant than the protein-coding *RASSF1A* and *RASSF1C* mRNAs, which are expressed from the same host locus (Figure S2E), demonstrating that *ANRASSF1* was expressed at low levels in the cell.

### *ANRASSF1* is transcribed from an independent promoter

A putative bidirectional promoter region spanning the transcription start-sites of *ANRASSF1* and *RASSF1C* has been predicted *in silico* [28]. We referred to the region on the genomic plus strand upstream of *ANRASSF1* as the “antisense promoter” and the region in the minus strand upstream of *RASSF1C* as the “sense promoter” (Figure S3A). The activity of both promoters was measured *in vitro*, and both promoters showed a significant induction of the firefly luciferase reporter relative to the control (*p*<0.01) (Figure S3B). This result confirmed the presence of a bidirectional promoter, specifically indicating promoter activity upstream of the *ANRASSF1* lncRNA, thus supporting an independent antisense transcriptional unit in this locus.

### Overexpression of *ANRASSF1* negatively modulates the protein-coding *RASSF1A* mRNA and decreases the RASSF1A protein

**Figure 3:**
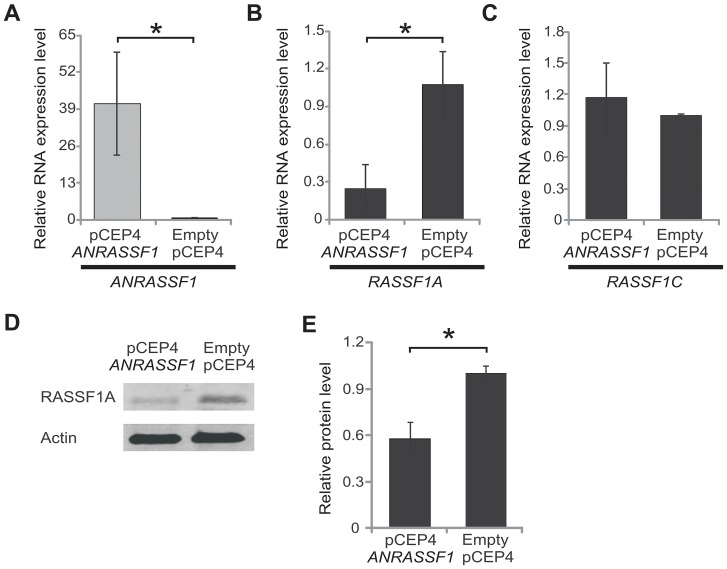
Overexpression of *ANRASSF1* decreases the protein-coding *RASSF1A* isoform. (A) Expression of *ANRASSF1* in HeLa cells transfected with the *ANRASSF1*-containing vector (pCEP4 *ANRASSF1*) compared with *ANRASSF1* expression in control HeLa cells transfected with an empty vector (empty pCEP4). Relative expression detected using RT-qPCR; these data are plotted relative to α-tubulin. These data show the means ± SD from three independent replicate transfection experiments. *p<0.01 relative to control. (B) Relative expression of *RASSF1A* isoform in *ANRASSF1*-transfected and control cells, as described in (A). These data show the means ± SD from three independent transfection experiments. *p<0.01 relative to control. (C) Relative expression of the *RASSF1C* isoform in *ANRASSF1*-transfected and control cells, as described in (A). These data show the means ± SD from three independent transfection experiments. (D) Western blot analysis using an antibody against RASSF1A protein in the lysates obtained from control HeLa cells (empty pCEP4) or *ANRASSF1*-transfected cells (pCEP4 *ANRASSF1*). An antibody against actin was used as control. (E) Densitometric analyses of the RASSF1A western blot signals from three replicate assays. Relative protein levels of RASSF1A normalized to actin. These data show the means ± SD from three independent transfection experiments. *p<0.02.

The observation of an inverse correlation between *ANRASSF1* and *RASSF1* expression (Figures 1D, 1E, 2 and S1) prompted us to determine whether *ANRASSF1* could act *in cis* to modulate the expression of the protein-coding gene in the same locus. We overexpressed *ANRASSF1* and subsequently measured the levels of the *RASSF1* transcript isoforms. When the expression levels of *ANRASSF1* were increased to 40-fold compared with the control cells (Figure 3A), *RASSF1A* expression was significantly decreased to 21% of its endogenous level (Figure 3B). Interestingly, the overexpression of *ANRASSF1* did not affect the abundance of the *RASSF1C* mRNA isoform (Figure 3C).

In parallel, the levels of RASSF1A protein were determined through western blot. Figure 3D shows that RASSF1A was reduced in pCEP4 *ANRASSF1*-transfected HeLa cell lines compared with control cells. Densitometric analysis of western blots from three replicates of cells overexpressing *ANRASSF1* showed a 57% decrease in the levels of RASSF1A protein compared with the normal levels observed in the control cells (Figure 3E).

### Overexpression of *ANRASSF1* increases the cell proliferation rate and decreases cell death

**Figure 4:**
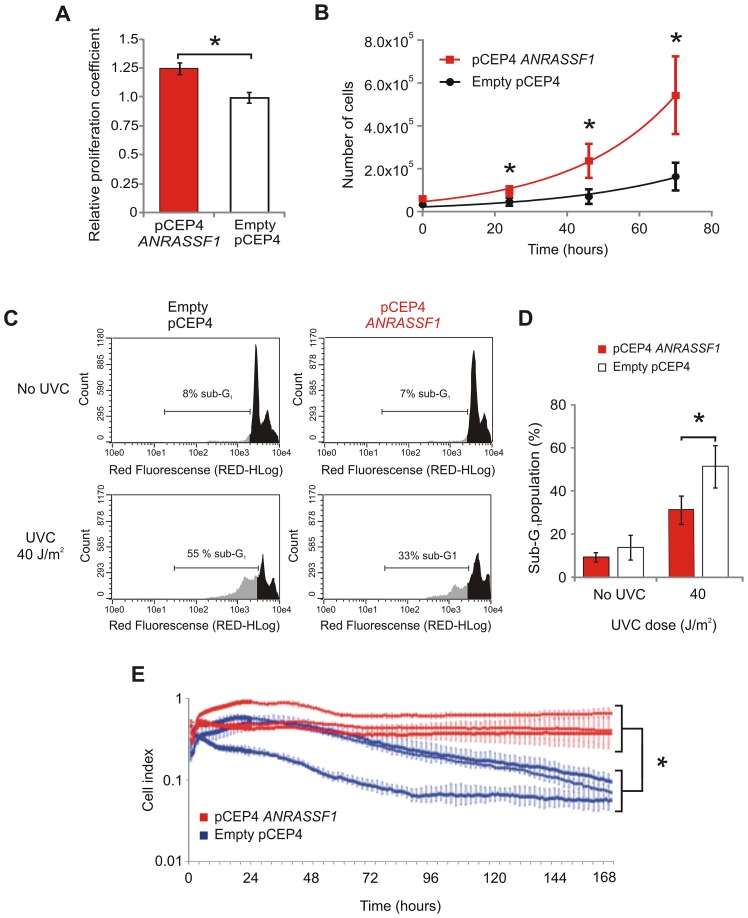
Overexpression of *ANRASSF1* increases cell growth and decreases UVC- and staurosporine-mediated cell death. (A) Cell proliferation coefficients of *ANRASSF1*-transfected cells (pCEP4 *ANRASSF1*) relative to control cells (empty pCEP4), as measured using MTS assay. These data show the means ± SD from three independent experiments. *t-test *p*<0.03 relative to control. (B) Effect of *ANRASSF1* overexpression on the cell population growth, as measured by counting the number of cells over time in culture and calculating the population doubling time. These data show the means ± SD from two independent experiments in triplicate. * *t*-test *p*<0.05. (C) DNA content histograms showing the effect of *ANRASSF1* overexpression on the cell cycle at 48 h after exposure to UVC irradiation (40 J/m^2^). The cells were labeled with propidium iodide. Light gray indicates the sub-G1 population. (D) Percent of sub-G_1_ population from experiments identical to (C). These data show the means ± SD from three independent replicate experiments. *t-test *p*<0.05 relative to control. (E) Effect of *ANRASSF1* overexpression on cell survival in the presence of the cytotoxic drug staurosporine (100 nM). The changes in impedance were measured using the xCELLigence system to continuously monitor cell attachment to the culture plates. Three independent biological replicates for either condition (pCEP4 *ANRASSF1* or empty pCEP4) are shown, each representing the means ± SD of two or three technical replicates; the red lines show cells overexpressing *ANRASSF1*, and the blue lines show control cells carrying empty pCEP4) vector. *t-test *p*<0.001, for the cell indices at 170 h.

To determine whether the decrease in RASSF1A protein due to overexpression of *ANRASSF1* would result in detectable phenotypic changes, cell-proliferation assays were performed. First, we observed that cells overexpressing *ANRASSF1* proliferated at a significantly faster rate than the control cells (Figure 4A), with a significant 22% average increase in the proliferation rate (*p*<0.03) determined using an MTS assay. We also measured cell population growth by directly counting the number of cells over time in culture in a Neubauer chamber (Figure 4B); the adjusted exponential growth functions (R^2^ ranging from 0.93 to 0.99 throughout the replicates) showed calculated doubling times of 20.2±1.9 and 25.0±3.7 h for the cells overexpressing *ANRASSF1* and the control cells, respectively, with a significant 24% average increase in the cell growth rate (*p*<0.05) upon *ANRASSF1* overexpression (Figure 4B). The 22 to 24% increase in cell proliferation rate observed in the MTS and cell number counting assays upon the increase in *ANRASSF1* expression, with a resulting decrease of RASSF1A abundance, is consistent with the tumor suppressor function of this protein. This increase is comparable to the 26% increase in cell proliferation over one day previously observed with *RASSF1A* siRNA [29].

In addition to reducing cell proliferation, *RASSF1A* exerts a proapoptotic signaling function [30]. We therefore measured the cell death caused by exposure to UVC light or the cytotoxic anti-cancer drug staurosporine to determine whether the overexpression of *ANRASSF1* and the resulting decrease in *RASSF1A* would affect the course of cell death. Figure 4C shows that UVC irradiation (40 J/m^2^) caused an important increase in cell death, as shown by the increase in the sub-G_1_ population of cells, and the overexpression of *ANRASSF1* substantially decreased the UVC-induced sub-G1 population, which is indicative of a reduction in cell death. Figure 4D shows the average increase in the sub-G_1_ population in the control cells (empty pCEP4) exposed to UVC compared with no-UV, as measured in three independent replicas, and a significant decrease in the percentage of the sub-G_1_ population in cells overexpressing *ANRASSF1* after exposure to UVC (*t*-test *p*<0.05), which is indicative of reduced cell death in the latter condition compared with the control (empty pCEP4) cell line.

Next, we measured cell death in response to the cytotoxic drug staurosporine (100 nM), a classical initiator of apoptosis by both caspase-dependent and caspase-independent pathways [31], and again tested the effect of *ANRASSF1* overexpression on cell death. We employed the xCELLigence platform microelectric assay based on the changing impedance of electrodes in the presence of live cells to examine the drug-induced cytotoxicity over an extended period of time in culture (170 h). Figure 4E shows that after exposure to staurosporine for 96 h, the cells overexpressing *ANRASSF1* were considerably more resistant to death, remaining attached to the culture plates, even after 170 h; the measured cell index, which corresponded with the number of live cells attached to the plate (Figure 4E), showed a significant 4.2-fold average increase in cells overexpressing *ANRASSF1* compared with the control cells (empty pCEP4) at 170 h in the three biological replicates (*p*<0.001). These findings are consistent with the proapoptotic signaling function of *RASSF1A* [30] and indicate that *RASSF1A* silencing through the overexpression of *ANRASSF1* attenuated the cell death signal induced through UVC irradiation or staurosporine treatment.

### *ANRASSF1* silencing positively modulates the protein-coding *RASSF1A* message levels

**Figure 5:**
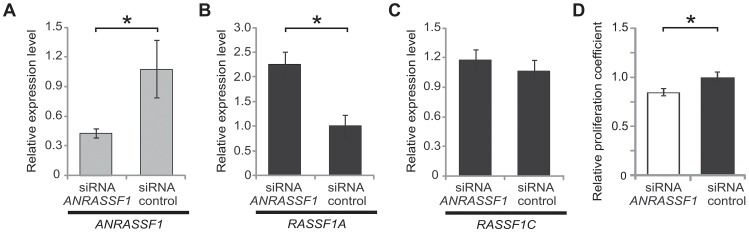
*ANRASSF1* knockdown increases the *RASSF1A* isoform expression and decreases cell proliferation. (A) Expression levels of *ANRASSF1* in cells treated with siRNA *ANRASSF1* relative to the expression of *ANRASSF1* in control cells treated with scrambled siRNA (siRNA control). The relative expression was detected using RT-qPCR normalized to α-tubulin. These data show the means ± SD from three independent experiments. *p<0.01 relative to control. (B) The relative expression levels of *RASSF1A* in siRNA *ANRASSF1* cells and siRNA control cells, as described in (A). These data show the means ± SD from three independent experiments. *p<0.01 relative to the control. (C) The relative expression levels of *RASSF1C* in siRNA *ANRASSF1* cells and siRNA control cells, as described in (A). (D) Cell proliferation coefficients of siRNA *ANRASSF1* cells relative to the control cells (siRNA control). These data show the means ± SD from three independent experiments. *p< 0.02 relative to control.

To further document the effect of *ANRASSF1* abundance on the expression of *RASSF1A*, we designed specific siRNAs to silence *ANRASSF1*. A pool of three different siRNAs targeting *ANRASSF1* significantly reduced *ANRASSF1* expression to 39% of its endogenous level compared with a scrambled siRNA control (Figure 5A). Concomitantly, we observed a 2.25-fold increase in the relative abundance of *RASSF1A* mRNA (Figure 5B). The decrease in *ANRASSF1* expression did not affect the abundance of the *RASSF1C* isoform (Figure 5C), which is consistent with the lack of effect of *ANRASSF1* overexpression on *RASSF1C* previously observed.

Next, we performed cell-proliferation assays to determine whether *ANRASSF1* knockdown and the consequent increase in the *RASSF1A* mRNA levels affected the cell proliferation rate. We observed that cells with silenced *ANRASSF1* expression proliferated at a significantly slower rate than control cells (Figure 5D), with an average 15% decrease in the proliferation rate compared with control cells (*p*<0.02).

### Accumulation of histone H3K27 trimethylation to the *RASSF1A* promoter locus is dependent on the level of *ANRASSF1* binding to PRC2

We sought to gain mechanistic insight into the involvement of the lncRNA *ANRASSF1* in regulating *RASSF1A* expression. *ANRASSF1* has nuclear localization, suggesting that this protein might be involved in the recruitment of other factors (MYC/PRC2) involved in *RASSF1A* gene silencing through H3K27 trimethylation and/or DNA promoter methylation [10].

**Figure 6:**
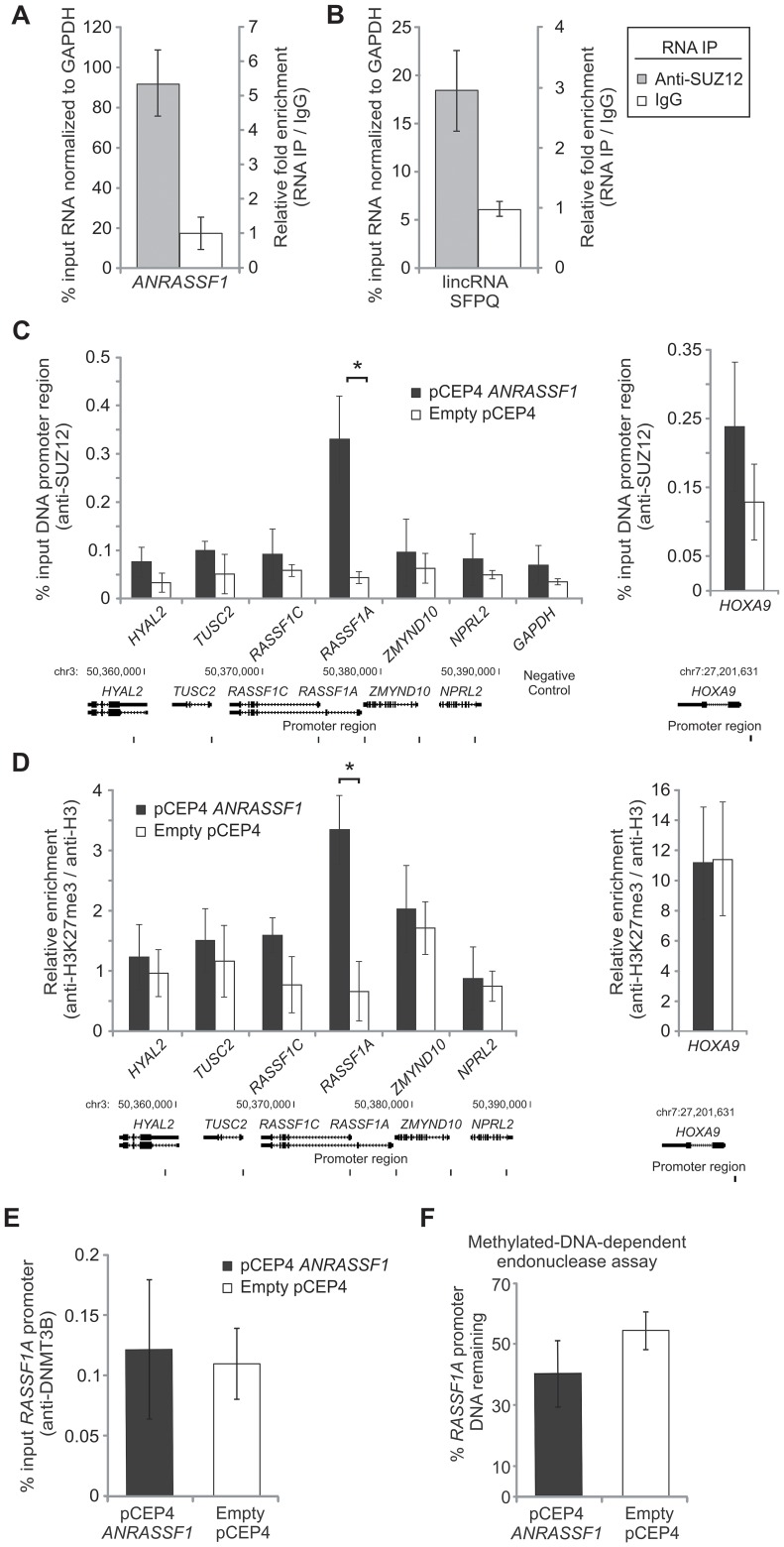
*ANRASSF1* interacts with PRC2 and affects its occupancy at the *RASSF1A* promoter. (A) Endogenous *ANRASSF1* levels bound to PRC2 were measured in HeLa cells through RNA IP with anti-SUZ12 relative to the input. A control IP with non-specific IgG was performed in parallel. As a negative control, *GAPDH* mRNA, which was not expected to bind to PRC2, was used. The percent input in the IP fractions was shown as the *ANRASSF1*/*GAPDH* ratio. These data show the means ± SD from three independent experiments. (B) lincRNA S*FPQ* is a positive control that binds to PRC2; RNA IP was assayed as in (A). These data show the means ± SD from three independent experiments. (C) ChIP assay using an anti-SUZ12 antibody in HeLa cells overexpressing *ANRASSF1* (pCEP4 *ANRASSF1*, black bars) or control cells (empty pCEP4, white bars). The promoter regions of the *RASSF1A* and *RASSF1C* isoforms and two other genes on either side of the *RASSF1* locus on chr 3 were investigated (the promoters are indicated with vertical lines in the scheme shown at the bottom of the figure). Control *GAPDH* was included as a gene not expected to be regulated through SUZ12. Control *HOXA9* is a gene regulated through SUZ12 [34] and encoded on chr 7. The amount of DNA in anti-SUZ12 samples at each promoter region detected through qPCR analysis was calculated in relation to the input. These data show the means ± SD from three independent experiments. *t-test *p*<0.02 relative to control at the *RASSF1A* locus. No significant changes were detected at other loci. (D) ChIP analysis using an anti-H3K27me3 antibody in an assay similar to that described in (C), except that the enrichment was calculated relative to anti-H3 ChIP. These data show the means ± SD from three independent experiments. *t-test *p*<0.02 relative to control at the *RASSF1A* locus. No significant changes were detected at the other loci. (E) ChIP analysis using an anti-DNMT3B antibody in an assay similar to that described in (C). These data show the means ± SD from three independent experiments. No significant change was observed. (F) DNA methylation at the *RASSF1A* promoter region was detected through qPCR with a methylation-dependent McrBC endonuclease assay in the *ANRASSF1*-overexpressing or control cells. The percentage of DNA remaining was calculated after comparing the amount of DNA amplified through qPCR following treatment with McrBC endonuclease with that following no-endonuclease treatment. These data show the means ± SD from three independent experiments. No significant change was observed.

First, we examined whether endogenous *ANRASSF1* was physically associated with PRC2 using a native non-cross linked RNA immunoprecipitation (RNA-IP) with an antibody specific to SUZ12, a member of the PRC2 complex. Figure 6A shows that the endogenous *ANRASSF1* was 5.3-fold enriched with respect to negative control RNA, which was not expected to bind PRC2, namely *GAPDH* mRNA. The endogenous lincRNA *SFPQ*, which binds PRC2, as determined with anti-SUZ12 and anti-EZH2 antibodies [18], was used as positive control and showed a 2.8-fold enrichment (Figure 6B).

Using an antibody specific to EZH2, another member of the PRC2 complex (Figure S4), we observed that endogenous *ANRASSF1* was enriched in the anti-EZH2 RNA-IP fraction relative to the input compared with the IgG fraction.

Next, we determined whether the PRC2 complex was bound to the promoter region of *RASSF1A* and if the overexpression of *ANRASSF1* affects PRC2 occupancy in this region. We performed chromatin immunoprecipitation (ChIP) using an anti-SUZ12 antibody and observed a significant 8-fold increase of the *RASSF1A* promoter DNA bound to PRC2 in cells overexpressing *ANRASSF1* compared with control cells expressing endogenous levels of *ANRASSF1* (Figure 6C). Interestingly, no significant change in PRC2 occupancy upon *ANRASSF1* overexpression was observed either at the promoter region of the *RASSF1C* isoform or the promoter of the four neighboring genes, two on either side at the *RASSF1 locus* (Figure 6C), including the two well-characterized tumor suppressor genes *TUSC2* [32] and *NPRL2* [33]. As additional controls, no significant change was detected at either the promoter of *GAPDH*, a gene not regulated through SUZ12, or the promoter of *HOXA9*, a gene regulated through SUZ12 [34] and encoded in chromosome 7, away from the *RASSF1* locus in chromosome 3. These results indicate a correlation between the higher levels of *ANRASSF1* and the higher PRC2 occupancy at the *RASSF1A* promoter.

PRC2 is a histone-modifying enzyme complex responsible for adding di- and trimethylation marks onto H3K27. We determined whether *ANRASSF1* overexpression, which increases PRC2 occupancy, also affected the level of H3K27me3 at the *RASSF1A* promoter region. Figure 6D shows that there was a 5.1-fold enrichment of the *RASSF1A* promoter DNA associated with histone H3K27me3 when *ANRASSF1* was overexpressed compared with control cells with endogenous levels of *ANRASSF1*, confirming that H3K27 trimethylation in the *RASSF1A* promoter is dependent on the level of *ANRASSF1* binding to PRC2. No significant changes in H3K27me3 were observed at either the promoter region of *RASSF1C* or the promoter region of the four other genes in the vicinity of *RASSF1* (Figure 6D). In addition, no change was detected at the promoter region of *HOXA9*. These results suggest highly location-specific epigenetic modulation.

Because DNA methylation of the *RASSF1A* promoter involves PRC2 and DNMT3B [10], we examined whether the increased recruitment of PRC2 through *ANRASSF1* overexpression also affected DNMT3B occupancy at the *RASSF1A* promoter or DNA methylation at the *locus*. Figure 6E shows that the DNMT3B occupancy at the *RASSF1A* promoter is not affected by *ANRASSF1* overexpression in HeLa cells. A methylation-dependent endonuclease assay showed no significant effect on the DNA methylation at the promoter region of *RASSF1A* in cells overexpressing *ANRASSF1* compared with the control (Figure 6F).

### *ANRASSF1* is bound to DNA and required for the PRC2 occupancy at the *RASSF1A* promoter

**Figure 7:**
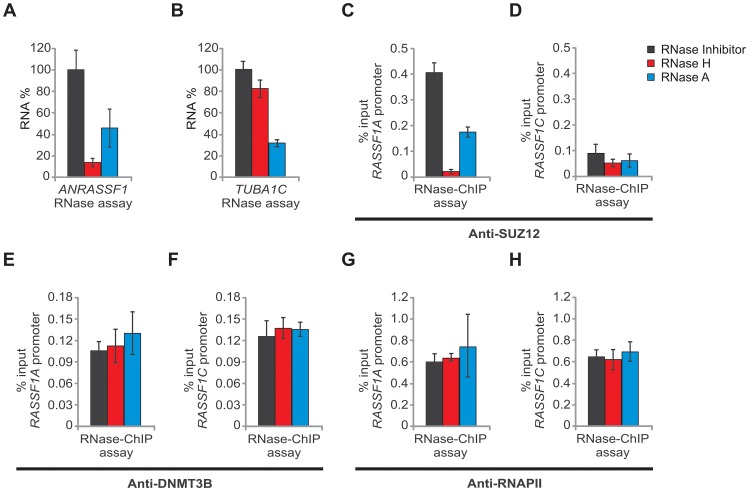
*ANRASSF1* mediates recruitment of SUZ12 to the *RASSF1A* promoter. (A) RNase assay for detection of *ANRASSF1* using RT-qPCR in permeabilized HeLa cells treated with RNase inhibitor (black bar), RNase H (red bar) or RNase A (blue bar). RNA% for each of the two RNase treatments was calculated relative to the corresponding values for the RNase inhibitor. These data show the means ± SD from three independent experiments. (B) As a control, alpha-tubulin mRNA was measured using RT-qPCR in parallel under the same conditions as described in (A). These data show the means ± SD from three independent experiments. (C) RNase-ChIP assay with anti-SUZ12 antibody in permeabilized HeLa cells treated with either RNase inhibitor (black bar), RNase H (red bar) or RNase A (blue bar). The amount of DNA at the *RASSF1A* promoter region detected through qPCR in anti-SUZ12 samples was calculated in relation to the input. These data show the means ± SD from two independent experiments that were performed in triplicate. (D) The amount of DNA at the *RASSF1C* promoter region was measured under the same conditions as described in (C). (E–F) The amount of DNA at the *RASSF1A* and *RASSF1C* promoter regions was measured under the same conditions as in (C–D), except that an anti-DNMT3B antibody was used. (G–H) The amount of DNA at the *RASSF1A* and *RASSF1C* promoter regions was measured under the same conditions as in (C–D), except that an anti-RNA Pol II antibody was used.

Recent reports have shown that lincRNAs, such as lncRNA_CCND1_ [35] and Mistral [36], bind to DNA and recruit proteins that act at the promoter regions of neighboring protein-coding genes to regulate their expression. We determined whether *ANRASSF1* might act in a similar manner through binding to DNA and recruiting the PRC2 complex in *cis* to the promoter of *RASSF1A*. For this purpose, we treated permeabilized HeLa cells with RNase H to deplete endogenous *ANRASSF1* associated with DNA, followed by ChIP using an anti-SUZ12 antibody. First, we measured the endogenous *ANRASSF1* levels in cells treated either with RNase inhibitor (Figure 7A, black bar) or RNase H (Figure 7A, red bar). With RNase H treatment, which digests RNA/DNA hybrids, the *ANRASSF1* level was reduced to 13%, suggesting that *ANRASSF1* is part of an RNA/DNA hybrid. Alpha-tubulin RNA, which was used as a control in this study, was only digested by RNase A (Figure 7B, blue bar), not RNase H (Figure 7B, red bar), as anticipated for an RNA that is not expected to form RNA/DNA hybrids. In parallel, we measured the PRC2 occupancy at the *RASSF1A* and *RASSF1C* promoter regions and observed that the maximum occupancy was obtained in the presence of RNase inhibitor (Figure 7C and 7D, black bars). Treatment with RNase H resulted in an almost total release of PRC2 from the *RASSF1A* promoter region (Figure 7C, red bar), while a low PRC2 occupancy at the *RASSF1C* promoter region (Figure 7D, black bar) and no significant reduction upon RNase H or RNase A treatment was observed (Figure 7D, red and blue bars); these data indicate that the PRC2 occupancy at the *RASSF1A* promoter region — not the *RASSF1C* promoter region — is driven by *ANRASSF1*.

We also performed an RNase-ChIP assay using an anti-DNMT3B antibody, and no differences in the occupancy at the *RASSF1A* or *RASSF1C* promoter regions were detected under RNase H or RNase A treatments (Figure 7E and 7F). This result indicates that the DNMT3B occupancy at the *RASSF1* locus is not driven by *ANRASSF1*; consistent with the observation described above, the DNMT3B occupancy at the *RASSF1A* promoter was not affected through *ANRASSF1* overexpression (Figure 6E).

Finally, a negative control RNase-ChIP with anti-RNA Pol II, a factor whose occupancy at the promoters of *RASSF1A* and *RASSF1C* is not expected to be affected by RNase H treatment, was included; treatment with RNase H or RNase A did not change the *RASSF1A* or *RASSF1C* promoter occupancy by RNA Pol II (Figure 7G and 7H), ruling out the notion that the effect on PRC2 would result from the toxic effect of RNase treatment on the cells.

Notably, in cells treated with RNase A, which digests single-stranded RNA (ssRNA), we observed both a reduction in the endogenous *ANRASSF1* (Figure 7A, blue bar) and the PRC2 occupancy at the *RASSF1A* promoter (Figure 7C, blue bar); however, the reduction was considerably smaller than that obtained with RNase H treatment (see Figure 7A and 7C, red bars), suggesting that the ssRNA in the nucleoplasm was in rapid equilibrium with the RNA/DNA hybrids and that a shift from hybrids to single-strand occurred as the hybrid was digested. Alternatively, there could be two distinct populations of ssRNA and RNA-DNA hybrids, and both could play a role in the association of PRC2 with chromatin.

A similar pattern of RNase H digestion and PRC2 occupancy at the *RASSF1A* promoter was observed in cells overexpressing *ANRASSF1* (Figure S5). Notably, the amount of *ANRASSF1* under overexpression conditions was 40-fold higher than that endogenously expressed (see Figure 3A); therefore, the similar percentage of *ANRASSF1* depletion achieved through RNase H treatment (Figure S5B) indicates that the absolute amount of the remaining *ANRASSF1* was also approximately 40-fold higher in overexpressing cells, resulting in a smaller reduction of PRC2 binding at the *RASSF1A* promoter in overexpressing cells treated with RNase H (45% reduction with respect to the inhibitor, Figure S5A) compared with cells with endogenous lncRNA treated with RNase H (90% reduction with respect to the inhibitor, Figure 7C).

**Figure 8:**
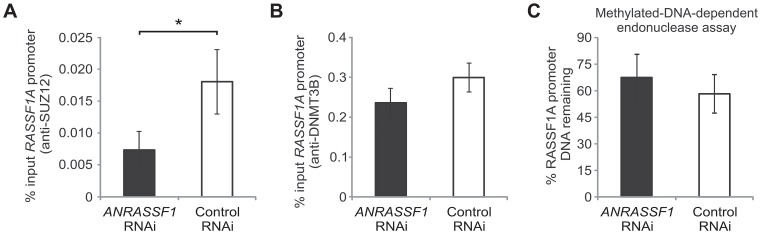
PRC2 is specifically directed by *ANRASSF1* lncRNA to the *RASSF1A* promoter. (A) ChIP assay using an anti-SUZ12 antibody in HeLa cells transfected with an RNAi oligonucleotide antisense to *ANRASSF1* or with a control scrambled oligonucleotide. The amount of DNA at the *RASSF1A* promoter region detected using qPCR in the anti-SUZ12 samples was calculated in relation to the input. These data show the means ± SD from two independent experiments performed in triplicate. *p<0.03 relative to control. (B) ChIP assay using an anti-DNMT3B antibody in an assay similar to (A). (C) DNA methylation at the *RASSF1A* promoter region was detected through qPCR in a methylation-dependent McrBC endonuclease assay in cells transfected with an RNAi oligonucleotide antisense to *ANRASSF1* or with a control scrambled oligonucleotide. The percent remaining DNA was calculated by comparing the amount of DNA at the promoter of *RASSF1A* amplified through qPCR following treatment with McrBC endonuclease against that amplified in the no-endonuclease treatment. These data show the means ± SD from three independent experiments. No significant change was observed.

RNAi was employed to determine whether the reduction of PRC2 occupancy at the *RASSF1A* promoter is specifically associated with *ANRASSF1* expression. The knockdown of *ANRASSF1* to 40% of the endogenous level (data not shown) caused a 55% reduction in PRC2 occupancy at the *RASSF1A* promoter (Figure 8A). Similarly, Palakurthy et al. [10] knocked down *EZH2* expression using RNAi and observed an increase in the expression of *RASSF1A*. Taken together, these results indicate that the recruitment of PRC2 complex to the promoter of *RASSF1A* specifically relies on the association of PRC2 with an *ANRASSF1*/DNA hybrid structure.

In parallel, we also measured the DNMT3B occupancy and levels of DNA methylation at the *RASSF1A* promoter (Figure 8B and 8C); neither condition was affected by *ANRASSF1* knockdown, which is consistent with our observations of a lack of effect on DNMT3B occupancy when *ANRASSF1* was either overexpressed or digested by RNase.

## Discussion

Some of the features identified for *ANRASSF1*, such as the presence of a 5′ cap, transcription through RNAPII, nuclear enrichment and binding to PRC2, are shared with other lncRNAs [18], [37]. However, the *ANRASSF1* half-life is short (∼50 min) compared with other moderately stable lncRNAs that exert epigenetic roles, such as *Air*, *Kcnq1ot1* and *Xist*, which have half-lives of 2.1, 3.4 and 4.6 h, respectively [38].

*ANRASSF1* is an unspliced intronic lncRNA and a member of a poorly characterized class of RNAs, as intronic unspliced RNAs are occasionally suspected as technical artifacts or transcriptional noise [39]. Notably, the intronic *ANRASSF1* in HeLa cells showed considerably lower expression levels compared with the well-studied intergenic lncRNAs (Figure S2). Nevertheless, changes in *ANRASSF1* abundance through ectopic overexpression or siRNA-mediated knockdown have specifically affected *RASSF1A* gene expression through PRC2 recruitment. Most importantly, the changes in *ANRASSF1* abundance did not affect the expression of *RASSF1C*, another mRNA isoform expressed in the *RASSF1 locus* under a different promoter, or the levels of H3K27me3 and PRC2 recruitment to the *RASSF1C* promoter and the promoters of neighboring genes in the *RASSF1 locus*, including two other well-characterized tumor suppressor genes, namely *TUSC2* [32] and *NPRL2* [33]. These data suggest a highly location-specific epigenetic regulation at the histone level that is driven by lncRNA *ANRASSF1*.

DNA methylation of the *RASSF1A* promoter involves PRC2 and DNMT3B DNA-methyl transferase in lung carcinoma cell lines that have high HOX3B expression [10]. We observed a limited, non-significant effect on DNA methylation at the *RASSF1A* promoter when the PRC2 occupancy was changed through *ANRASSF1* overexpression or knockdown in HeLa cells. Other factors, such as HOXB3 expression and DNMT3B occupancy at the *RASSF1A* promoter, are involved in DNA methylation [10], and the observed limited increase in DNA methylation in HeLa cells overexpressing *ANRASSF1* might reflect the eventual limited availability of these factors in HeLa cells. Indeed, the promoter region of *RASSF1A* in HeLa cells is hypomethylated [3].

Recently, many intronic RNA sequences capable of binding the PRC2 core component EZH2 have been detected in human cancer cells [20]. These authors characterized an EZH2-bound intronic unspliced RNA for the methyltransferase gene *SMYD3* in more detail and observed that EZH2 binds to sense intronic ncRNA either at the pre-mRNA stage or after splicing and intron removal [20]. The overexpression of sense ncRNA from the *SMYD3* locus caused the epigenetic in *cis* regulation of *SMYD3* and a decrease in cell proliferation [20]. Different from *ANRASSF1*, this intronic unspliced lncRNA is not derived from an independent transcriptional unit in the locus.

Another example of intronic unspliced lncRNA recruiting PRC2 is *Kcnq1ot1*, which is transcribed from the *Kcnq1* locus. The lncRNA *Kcnq1ot1* regulates the expression of ten genes at the *Kcnq1* imprinted cluster [40] through a mechanism clearly distinct from the location-specific *cis*-acting regulation of the *RASSF1A* isoform observed here.

**Figure 9:**
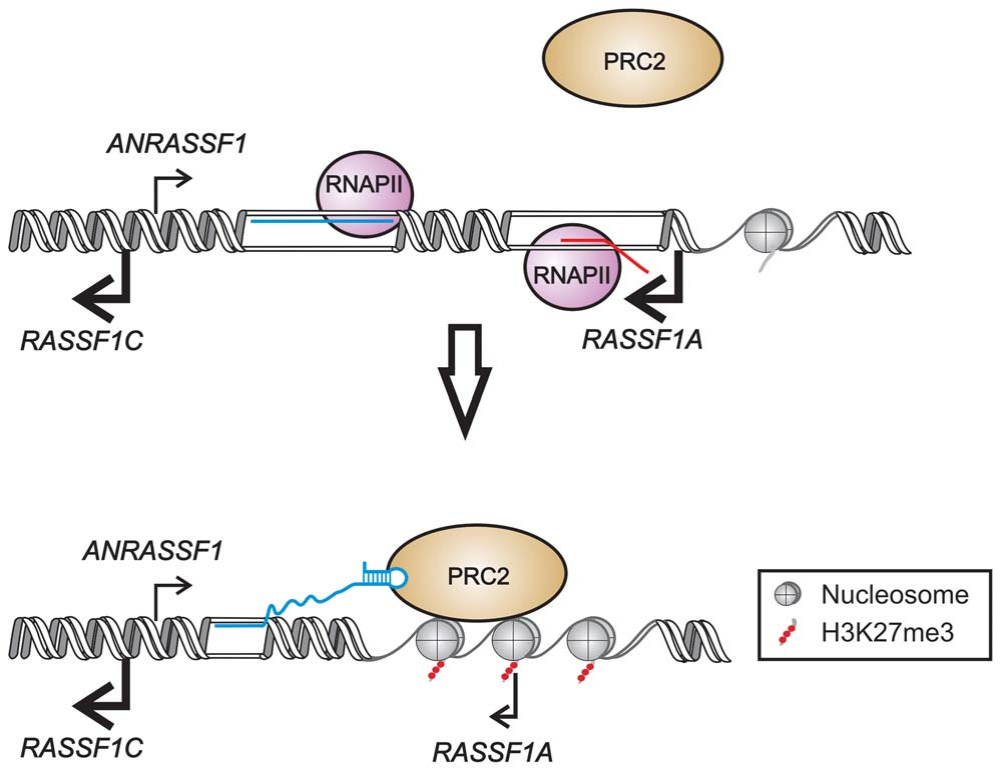
Proposed model for *ANRASSF1* function at the *RASSF1* genomic *locus*. We postulate that lncRNA *ANRASSF1* (blue line) interacts with genomic DNA at the transcription site, forming an RNA/DNA hybrid, and recruits the chromatin-modifying PRC2 complex to the protein-coding *RASSF1A* gene promoter region. The recruitment of the PCR2 complex results in the selective modification of the histone H3K27 pattern of methylation (red circles) at the *RASSF1A* promoter, leading to a specific reduction in *RASSF1A* transcriptional activity with no effect on the *RASSF1C* transcription.

In the present study, we demonstrated that the unspliced intronic *ANRASSF1* binds to PRC2 and is required for PRC2 occupancy at the *RASSF1A* promoter region. We postulate an *in cis* mechanism (Figure 9) by which the interaction of *ANRASSF1* with both DNA at its transcription site and PRC2 induces the recruitment of PRC2 to the *RASSF1A* and not the *RASSF1C* promoter. In turn, PRC2 recruitment induces the accumulation of the repressive mark H3K27me3, which culminates in the transcriptional down-regulation of only the *RASSF1A* isoform (Figure 9). This model, which involves a lncRNA/DNA hybrid, would be analogous to that of the intergenic lncRNA *Mistral*; however, different from the model proposed here, the intergenic lncRNA *Mistral* mediates the activation of *HOXA6* and *HOXA7* transcription *in trans* [36]. The MLL1 SET domain is involved in recognizing and binding the *Mistral*/DNA hybrid, thus triggering dynamic changes in the chromosome conformation [36] that involve the formation of a loop in the DNA at the promoter of the *HOXA6* and *HOXA7* neighboring genes. A loop conformation at the *RASSF1A* promoter, similar to that proposed for the *Mistral* locus, could be the subject of further investigation.

*ANRASSF1* is one of the thousands of unspliced lncRNAs, named TIN and PIN RNAs, transcribed from the intronic regions of 74% of the protein-coding genes in the human genome [22], [41]. The formation of an RNA/DNA hybrid at the transcription locus could result in a highly location-specific effect for a number of these unspliced intronic lncRNAs. Indeed, 141 lncRNAs map to the intronic regions of 127 protein-coding genes and are associated with chromatin, as identified through chromatin-RNA isolation and high-throughput sequencing [42]; of these, 52% are TIN/PIN RNAs [42]. We hypothesize that other non-characterized unspliced intronic lncRNAs transcribed in the human genome might contribute to a diverse location-specific epigenetic modulation at the loci where they are transcribed [43].

There is increasing evidence that lncRNAs are involved in a number of human diseases [44]–[46], particularly in cancer [46]–[50]. Taken together, our results reveal a novel mechanism for epigenetic regulation at the *RASSF1* locus that involves the antisense unspliced lncRNA *ANRASSF1*, suggesting an inverse correlation between *ANRASSF1* and *RASSF1A* expression in both tumor and non-tumor cell lines. Further studies on the potential involvement of *ANRASSF1* expression in tumorigenesis are warranted. These results could be the tip of the iceberg of an epigenetic modulation mechanism driven through unspliced intronic lncRNAs that might act at highly gene-specific loci in the human genome.

## Materials and Methods

### Cell lines and RNA extraction

All cell lines were obtained from the American Type Culture Collection (ATCC) and grown as recommended in media supplemented with 10% fetal bovine serum and 1% penicillin/streptomycin (Gibco). Total RNA was extracted using Trizol (Invitrogen) and purified using the RNAspin Mini kit (GE Healthcare) according to the manufacturer's instructions, except for an extended 1 h treatment with DNase I. Total RNA was quantified on a NanoDrop ND-1000 Spectrophotometer (NanoDrop Technologies). Total RNA integrity was assessed using an Agilent 2100 Bioanalyzer (Agilent Technologies).

### RACE and primer-walking PCR

For RACE-PCR, we used a commercial Human Colon Marathon-Ready cDNA library (Clontech) prepared from poly(A) RNA and Advantage 2 polymerase (Clontech) according to the manufacturer's instructions. Primer-walking PCR, using the sequence obtained from the *in silico* prediction of the transcriptional start site (TSS), was performed as described in the supporting information (Protocol S1). All primers are listed in Table S1. The PCR products were sequenced using the Sanger method. The *ANRASSF1* full-length sequence has been deposited in GenBank under accession number KC330992.

### Strand specific RT–PCR

Orientation-specific RT-PCR was performed with 1.5 µg total RNA using a gene-specific primer complementary to the antisense or sense strand of *ANRASSF1* according to the recommendations of Super Script III kit protocol (Invitrogen). Subsequently, PCR was performed using the internal primer pair indicated in Figure 1B. The primers are listed in Table S1. To control for DNA contamination in the RNA sample, reverse transcription with no RT-primer was performed, followed by PCR.

### Reverse transcription and quantitative real-time PCR (RT-qPCR)

Oligo-dT-primed reverse transcription (RT) was performed using 1 µg of total RNA according to the Super Script III kit protocol (Invitrogen). The relative levels of the *ANRASSF1* and *RASSF1* isoforms and other lncRNAs were determined through quantitative real-time PCR (qPCR) (primers are shown in Table S2) with Power SYBR Green (Applied Biosystems) using the 7500 Real Time PCR System (Applied Biosystems). The levels of these transcripts were normalized to the level of tubulin and represented as a fold-change using the delta Ct method [51].

### RNA-seq

Poly(A)^+^-RNA was extracted from LNCaP cells in culture using the FastTrack MAG Maxi mRNA Isolation Kit (Invitrogen) according to the manufacturer's instructions with the following modifications: treatment with 25 U of amplification grade DNase I (Invitrogen) for 1 h at room temperature. RNA was quantified using the Quant-iT RiboGreen RNA Reagent (Invitrogen) and assessed for integrity through electrophoresis with the Bioanalyzer RNA Pico LabChip (Agilent Technologies). Poly(A)^+^-RNA from three biological replicates was processed for Illumina sequencing using the standard protocol for strand-oriented paired-end 75-nt read sequencing, and a total of ∼430 million reads were obtained and mapped with TopHat [52] to the hg19 version of the human genome, followed by an ab-initio assembly using the Cufflinks tool [53], with the default parameters.

### Meta-analysis of the Affymetrix microarray expression data

A meta-analysis was performed using the publicly available microarray expression data from the Affymetrix HG-U133 Plus2 platform. The purification strategy, RNA processing method and hybridization strategy have been described in the original publications. The expression and sample annotation data were downloaded from the NCBI GEO website: GSE5823 [54], GSE11118 [55], GSE10879 [56], GSE12056 [57] and GSE13471 [58]. A Pearson correlation analysis between the expression signals of probe sets 240278_at (*ANRASSF1*) and 204346_s_at (*RASSF1* entire *locus*) was used in each study.

### *ANRASSF1* overexpression and silencing

For overexpression, the *ANRASSF1* cDNA was amplified through PCR (primers in Table S1), inserted into the pCEP4 expression vector (Invitrogen) to generate pCEP4 *ANRASSF1* and subsequently sequenced. The cells were transfected with a linearized pCEP4 *ANRASSF1* or a linearized empty vector (*Nhe*I) using FuGENE 6 Transfection Reagent (Roche). After transfection, the resistant cells were selected using 100 µg/mL hygromycin B (Invitrogen). For silencing, the HeLa cells were plated on 60-mm plates in medium without FBS. Twenty-four hours after plating, a pool of three distinct 25-mer siRNAs (5 nM each, final concentration) targeting *ANRASSF1* or a pool of three 25-mer scrambled siRNAs (Invitrogen) (Table S3) were transfected using Lipofectamine RNAimax (Invitrogen). The total RNA was extracted at 48 h after transfection. Alternatively, 120 nM of a modified 20-mer oligo (Table S3) was used for *ANRASSF1* silencing in the ChIP assays, which were performed at 24 h after transfection using Lipofectamine RNAimax (Invitrogen).

### Western blotting

The cells were washed with PBS, lysed on ice in 20 mM imidazole (pH 7.2) containing 1 mM EDTA and 250 mM sucrose with complete protease inhibitor cocktail (Roche) and sonicated. The protein content in the lysates was determined using the BCA protein assay (Bio-Rad). Equal protein amounts (40 µg) were resolved through SDS-PAGE. The primary antibodies were anti-*RASSF1A* (Abcam – ab23950) and anti-actin (Millipore – mab1501); and secondary antibodies were labeled with Alexa Fluor 680 (Invitrogen). The signals were detected using an Odyssey Infrared Imaging System (LI-COR Biosciences). The signal intensities were quantified using Odyssey Application Software v3 (LI-COR Biosciences).

### Cell proliferation assay

HeLa cells transfected with empty pCEP4 or pCEP4 *ANRASSF1* were seeded onto 96-well plates. Each pair of biological replicates was seeded onto the same plate. Cell proliferation was evaluated in an MTS assay by measuring formazan absorbance at 24 and 48 h using the CellTiter 96 Aqueous One Solution Cell Proliferation Assay (Promega) in a SpectraMax Paradigm (Molecular Devices). Three independent replicate transfections were tested. The proliferation coefficient was defined as the ratio between the average measurements at 24 and 48 h. For the RNAi assay, HeLa cells were seeded onto 96-well plates in media without FBS. The transfections were performed at 24 h after the seeding, and the cells were evaluated for proliferation at 24 and 48 h, as described above.

### Population doubling time

To measure the effect of *ANRASSF1* overexpression on the cellular proliferation phenotype, 2×10^4^ cells overexpressing *ANRASSF1* or control cells were seeded onto a 6-well plate. The cells were trypsinized every 24 h for 5 days and counted on a Neubauer chamber. The adjustment of exponential functions to the curves and statistical analyses were performed using GraphPad Prism software.

### Analyses of the sub-G1 population after UV irradiation

To measure the UV-induced sub-G1 population, which is indicative of cell death, plates containing pre-seeded cells at 70% confluence were washed with PBS and irradiated with 40 J/m^2^ UVC light with a germicidal lamp (primary emission at 254 nm) at a rate of 1.0 J.m^−2^.s^−1^, as measured with a VLX 3W radiometer (Vilber Lourmat). After 48 h, the supernatant and the attached cells were collected and fixed with 70% ethanol. Staining with propidium iodide (PI) was performed at room temperature for 1 h in PBS solution containing 20 µg/mL PI (Sigma-Aldrich), 200 µg/mL RNase A (Invitrogen) and 0.1% Triton X-100 (Sigma-Aldrich). These samples were loaded onto a Guava PCA-96 System cytometer (Millipore), and the analyses were performed using CytoSoft software (Millipore).

### Staurosporine-induced cytotoxicity assay

The cytotoxicity induced through staurosporine treatment was evaluated with a real time cytotoxicity assay using the xCELLigence system (Roche). Briefly, 2×10^3^ cells overexpressing *ANRASSF1* or control cells were seeded in triplicate onto the wells of a 96-well E-plate (Roche). After 18 h, these samples were treated with 100 nM staurosporine (Sigma) or mock-treated with the same concentration of DMSO (zero time). Thereafter, the impedance was continuously measured according to the manufacturer's instructions and as previously described [59]. Three independent biological replicate experiments were performed, each with technical triplicates.

### RNA Immunoprecipitation (RIP)

Native, non-cross linked RIP was performed using the Magna RIP RNA-Binding Protein Immunoprecipitation Kit (Millipore) according to the manufacturer's instructions. The following antibodies were used from Millipore: Normal Mouse IgG (12-371) and anti-SUZ12 (03-179); and Abcam: anti-EZH2 (ab3748). The RNAs were extracted using Trizol, treated with TURBO DNase (Ambion) at 37°C for 30 min, purified using an RNeasy Micro Kit (Qiagen) and quantified with RiboGreen (Invitrogen). All RIP assays were performed in biological triplicate and were detected by RT-qPCR (primers in Table S2).

### Chromatin Immunoprecipitation (ChIP)

ChIP was performed using the EZ-Magna ChIP Chromatin Immunoprecipitation Kit (Millipore). The following antibodies were used from Millipore: normal mouse IgG (12-371), normal rabbit IgG (12-370) and anti-SUZ12 (03-179); and Abcam: anti-H3K27me3 (ab6002), anti-H3 (ab1791) and anti-DNMT3B (ab2851). The DNA was detected through qPCR (primers in Table S2).

### RNase ChIP

Permeabilization of HeLa cells and RNase treatment were performed as previously described [36]. After the RNase treatment, an aliquot (70% vol) was processed as described above in the ChIP protocol using the following antibodies from Millipore: normal mouse IgG (12-371), anti-SUZ12 (03-179) and anti-RNA Pol II clone CTD4H8 (05-623B); and Abcam: anti-DNMT3B (ab2851). The remaining aliquot (30% vol) was processed as described in the RNA extraction protocol and analyzed through RT-qPCR (the primers are listed in Table S2).

### DNA methylation assay

DNA was extracted using phenol/chloroform and Proteinase K using a previously published method [60] and fragmented through sonication. A 1-µg sample of this DNA was treated with 100 U of methylation-dependent McrBC endonuclease (New England Biolabs) at 37°C for 16 h. The amount of *RASSF1A* promoter DNA was measured using qPCR (primers in Table S2). As a control, an assay with no endonuclease was run in parallel. Three biological replicates were tested.

## Supporting Information

Figure S1 Negative correlation between *ANRASSF1* and *RASSF1* expression levels in cell lines and human tissues. Meta-analysis results from (A) MCF-7 cells, wild-type and estrogen-deprived cells from GSE10879 [56]; (B) K562 cells, wild-type and CREB-knockdown cells from GSE12056 [57]; and (C) colon tumor, normal colon and normal liver human tissues from GSE13471 [58].
(TIF)

Figure S2 *ANRASSF1* lncRNA is RNAPII-encoded and nuclear-enriched and has a short half-life. (A) *ANRASSF1* is transcribed through RNA Polymerase II (RNAPII). HeLa cell cultures were treated with the RNAPlI inhibitor α-amanitin (black) or with vehicle (mock, gray) for 24 h. The *ANRASSF1* transcript abundance before and after α-amanitin treatment was measured with RT-qPCR. The results were normalized using the level of pre-tRNA^Tyr^, transcribed through RNAPIII and plotted relative to the mock condition. RNAPII-transcribed α-tubulin is shown as a positive control. These data show the means ± SD from three independent experiments. *p<0.0001. (B) Presence of a 5′-end cap modification in *ANRASSF1*. Total RNA from HeLa cells was digested using the terminator 5′-phosphate-dependent exonuclease (5′ exo) alone or in combination with the tobacco acid pyrophosphatase (TAP) as indicated. Subsequently, the samples were reverse transcribed and used as a template for qPCR with primers for *ANRASSF1*, *α-tubulin* (positive control) or snRNA *U15A*, which does not have a 5′cap (negative control), as indicated. (C) Decay of *ANRASSF1* RNA in the presence of actinomycin D. HeLa cells were treated with the transcriptional inhibitor actinomycin D or vehicle for 0,1, 3, 6 and 8 h. At each time point, total RNA was isolated, and the *ANRASSF1* and *c-Myc* levels were measured using RT-qPCR, and normalized to that of an untreated sample. These data show the means ± SD from two independent experiments in triplicate. The insets show parameters for the fitted curves using one-phase exponential decay. (D) The relative abundance of the *ANRASSF1* RNA transcript in the nuclear or cytoplasmic compartments. Cellular fractions enriched in the nuclear or cytoplasmic RNAs were prepared from HeLa cells. Comparable starting amounts of RNA from each fraction were used for oligo-dT primed reverse transcription reactions, followed by qPCR with primers for *ANRASSF1*. These data show the means ± SD from two independent experiments in triplicate. (E) Expression levels of some selected abundant intergenic lncRNAs and *RASSF1* main isoforms relative to *ANRASSF1*. Gene expression was measured in HeLa cells through RT-qPCR and normalized to *ANRASSF1*. These data show the means ± SD from three independent experiments.
(TIF)

Figure S3 A putative *ANRASSF1* promoter region shows activity in the luciferase assays. (A) Genomic localization of the constructs spanning the putative antisense promoter region of *ANRASSF1* (antisense promoter, solid gray DNA on the plus strand) and the putative sense promoter region of *RASSF1C* (sense promoter, solid gray DNA on the minus strand) used in the promoter activity luciferase assays. The arrows indicate the orientation of the *ANRASSF1* lncRNA and the protein-coding transcripts in the locus. (B) Promoter activity measured using the firefly luciferase assay. HeLa cells were transfected with pGL3 vectors harboring different constructs upstream of the firefly luciferase gene as indicated. Cells transfected with pGL3 empty (negative control) or pGL3 SV40 promoter plasmids (positive control) were assayed in parallel. These data show the means ± SD from three independent experiments. *p<0.01.
(TIF)

Figure S4 Endogenous *ANRASSF1* interaction with PRC2. (A) Endogenous levels of *ANRASSF1* bound to PRC2 were measured in HeLa cells through RNA IP using an anti-EZH2 antibody, and the results were referred to as % input. The IgG from non-immunized mouse was included as a control. The asterisk indicates non-detectable amounts. (B) The level of lincRNA *SFPQ*, which does not bind to the PRC2 complex, as determined using anti-SUZ12 and anti-EZH2 antibodies [18], was measured as a positive control. These data show the means ± SD from three independent experiments.
(TIF)

Figure S5 *ANRASSF1* mediates the recruitment of SUZ12 to the *RASSF1A* promoter region in HeLa cells overexpressing *ANRASSF1*. (A) RNase-ChIP assay for the *RASSF1A* promoter region measured through qPCR in DNA immunoprecipitated using an anti-SUZ12 antibody in permeabilized HeLa cells overexpressing *ANRASSF1*. The cells were treated with either RNase inhibitor (black bar), RNase H (red bar) or RNase A (blue bar). The amount of DNA at the *RASSF1A* promoter region detected through qPCR in anti-SUZ12 samples was calculated in relation to the input. These data show the means ± SD from two independent experiments in triplicate. (B) Detection of *ANRASSF1* using RT-qPCR with samples obtained from HeLa cells overexpressing *ANRASSF1* and previously permeabilized and treated with RNase inhibitor (black bar), RNase H (red bar) or RNase A (blue bar). The RNA% for each of the two RNase treatments was expressed relative to the corresponding values for RNase inhibitor. These data show the means ± SD from three independent experiments. (C) As a control, *alpha-tubulin* mRNA was measured in parallel with RT-qPCR under the same conditions as described in (B). These data show the means ± SD from three independent experiments.
(TIF)

Protocol S1 Materials and methods for RACE and primer-walking PCRs, 5′-cap analysis, RNA polymerase transcription inhibition, cell fractionation and the promoter assay.
(DOC)

Table S1 Primers used for strand-specific RT, cloning, 5′-cap assay, RACE and primer walking.
(DOC)

Table S2 Primers used for qPCR, ChIP, RIP, RNase-ChIP and methylation assays.
(DOC)

Table S3 Oligonucleotide sequences used for knockdown assays.
(DOC)
