# Supplementary figures and images for: The Intronic Long Noncoding RNA ANRASSF1 Recruits PRC2 to the RASSF1A Promoter, Reducing the Expression of RASSF1A and Increasing Cell Proliferation
Source: PLoS Genet. 2013 Aug 22;9(8):e1003705. doi: 10.1371/journal.pgen.1003705 (PMC3749938; doi:10.1371/journal.pgen.1003705)

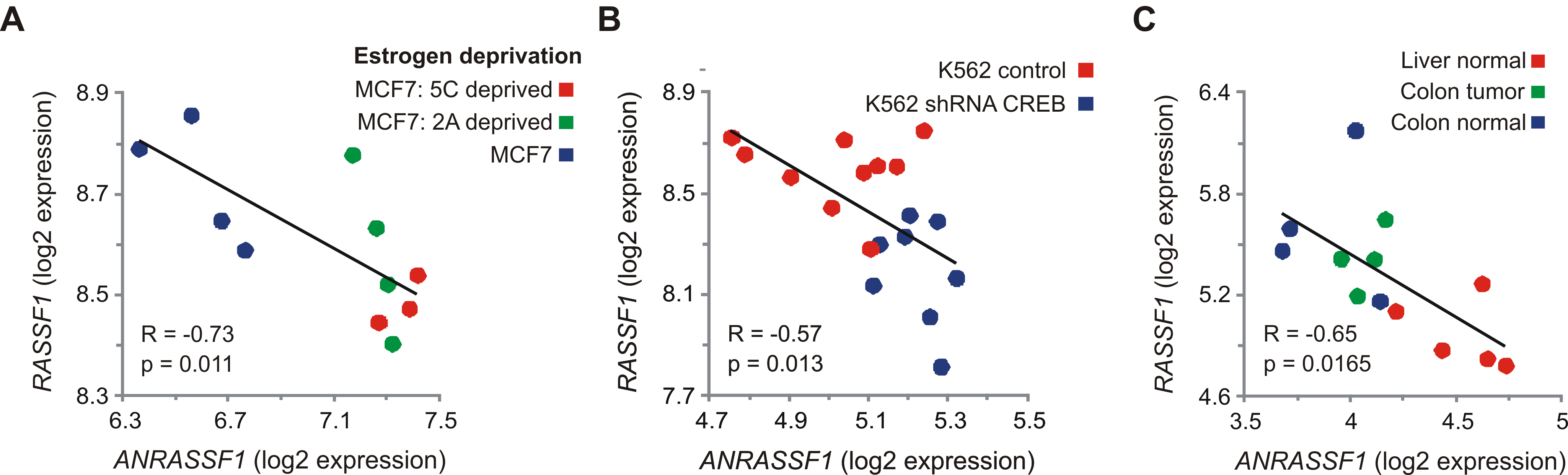

Supplement: Figure S1 — Negative correlation between ANRASSF1 and RASSF1 expression levels in cell lines and human tissues. Meta-analysis results from (A) MCF-7 cells, wild-type and estrogen-deprived cells from GSE10879 [56]; (B) K562 cells, wild-type and CREB-knockdown cells from GSE12056 [57]; and (C) colon tumor, normal colon and normal liver human tissues from GSE13471 [58]. (TIF) [file pgen.1003705.s001.tif]

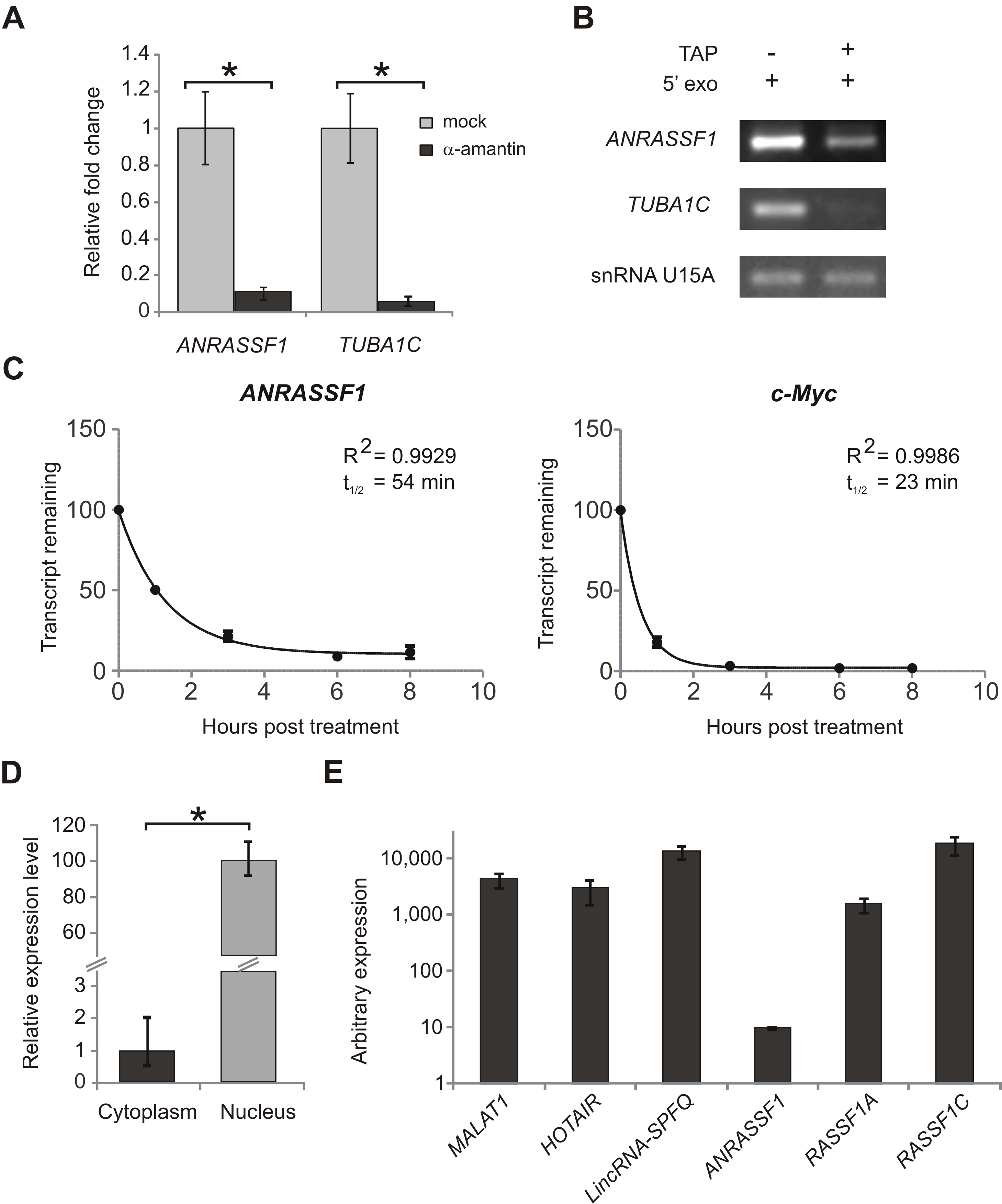

Supplement: Figure S2 — ANRASSF1 lncRNA is RNAPII-encoded and nuclear-enriched and has a short half-life. (A) ANRASSF1 is transcribed through RNA Polymerase II (RNAPII). HeLa cell cultures were treated with the RNAPlI inhibitor α-amanitin (black) or with vehicle (mock, gray) for 24 h. The ANRASSF1 transcript abundance before and after α-amanitin treatment was measured with RT-qPCR. The results were normalized using the level of pre-tRNATyr, transcribed through RNAPIII and plotted relative to the mock condition. RNAPII-transcribed α-tubulin is shown as a positive control. These data show the means ± SD from three independent experiments. *p<0.0001. (B) Presence of a 5′-end cap modification in ANRASSF1. Total RNA from HeLa cells was digested using the terminator 5′-phosphate-dependent exonuclease (5′ exo) alone or in combination with the tobacco acid pyrophosphatase (TAP) as indicated. Subsequently, the samples were reverse transcribed and used as a template for qPCR with primers for ANRASSF1, α-tubulin (positive control) or snRNA U15A, which does not have a 5′cap (negative control), as indicated. (C) Decay of ANRASSF1 RNA in the presence of actinomycin D. HeLa cells were treated with the transcriptional inhibitor actinomycin D or vehicle for 0,1, 3, 6 and 8 h. At each time point, total RNA was isolated, and the ANRASSF1 and c-Myc levels were measured using RT-qPCR, and normalized to that of an untreated sample. These data show the means ± SD from two independent experiments in triplicate. The insets show parameters for the fitted curves using one-phase exponential decay. (D) The relative abundance of the ANRASSF1 RNA transcript in the nuclear or cytoplasmic compartments. Cellular fractions enriched in the nuclear or cytoplasmic RNAs were prepared from HeLa cells. Comparable starting amounts of RNA from each fraction were used for oligo-dT primed reverse transcription reactions, followed by qPCR with primers for ANRASSF1. These data show the means ± SD from two independent experiments in tri [file pgen.1003705.s002.tif]

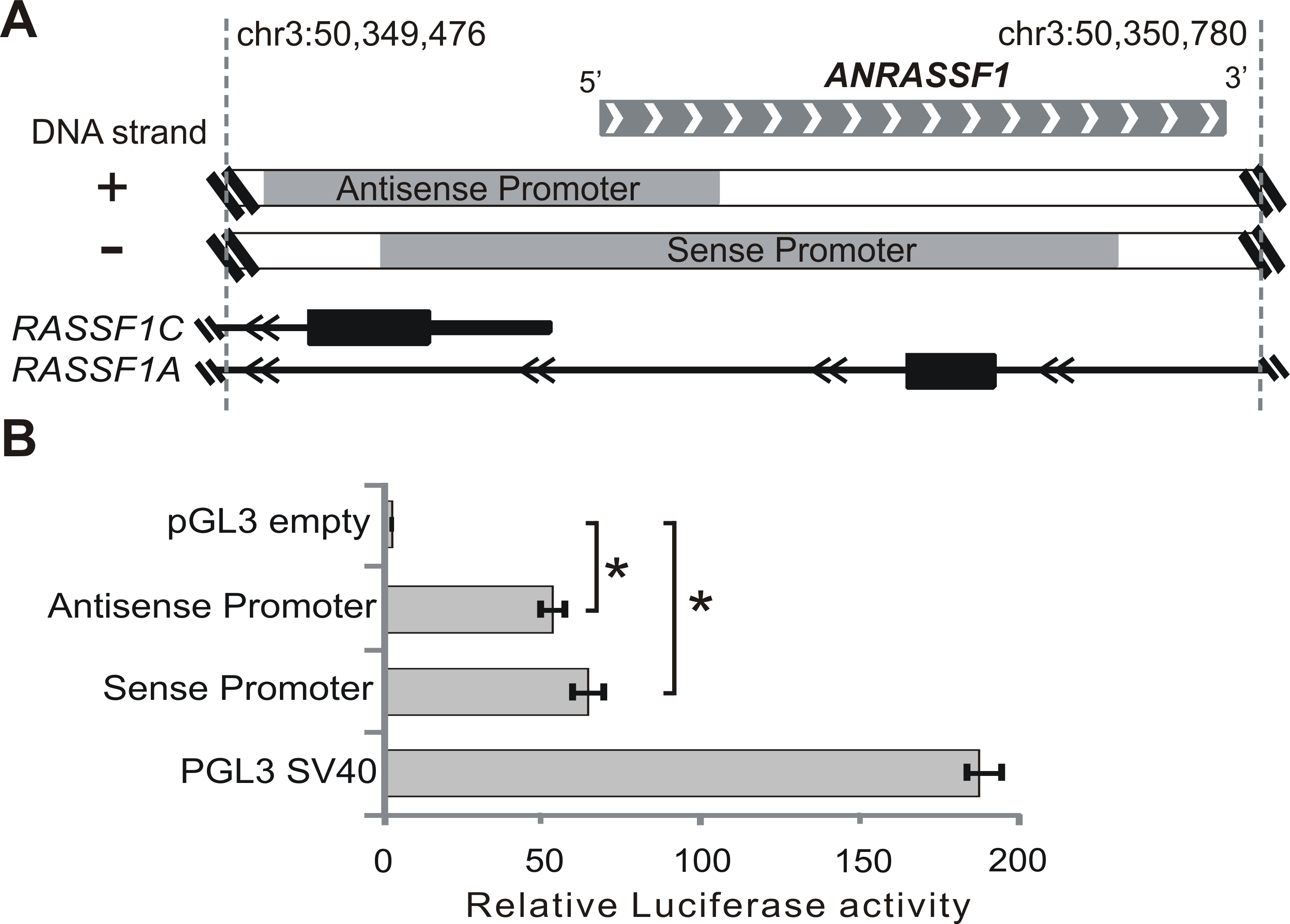

Supplement: Figure S3 — A putative ANRASSF1 promoter region shows activity in the luciferase assays. (A) Genomic localization of the constructs spanning the putative antisense promoter region of ANRASSF1 (antisense promoter, solid gray DNA on the plus strand) and the putative sense promoter region of RASSF1C (sense promoter, solid gray DNA on the minus strand) used in the promoter activity luciferase assays. The arrows indicate the orientation of the ANRASSF1 lncRNA and the protein-coding transcripts in the locus. (B) Promoter activity measured using the firefly luciferase assay. HeLa cells were transfected with pGL3 vectors harboring different constructs upstream of the firefly luciferase gene as indicated. Cells transfected with pGL3 empty (negative control) or pGL3 SV40 promoter plasmids (positive control) were assayed in parallel. These data show the means ± SD from three independent experiments. *p<0.01. (TIF) [file pgen.1003705.s003.tif]

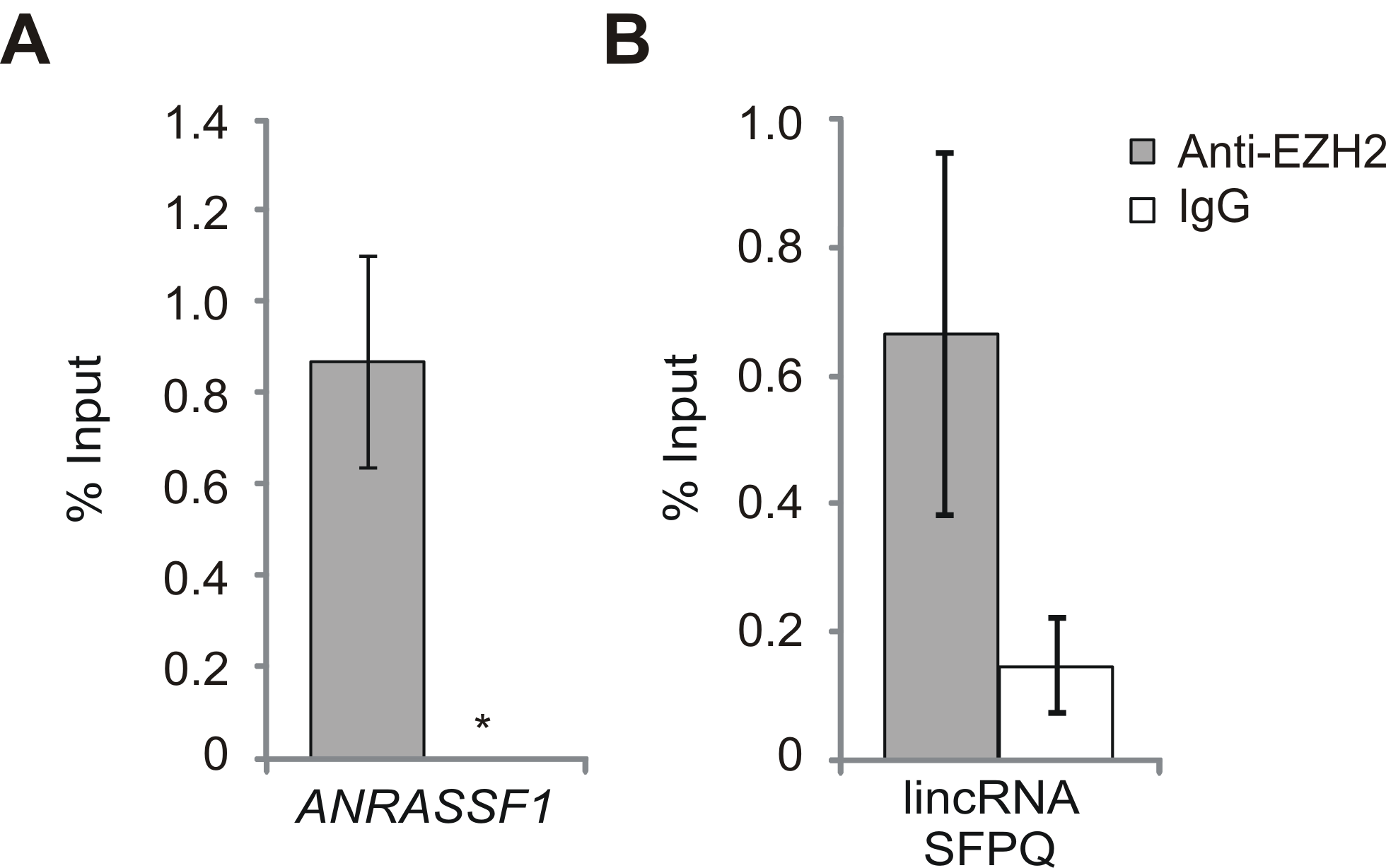

Supplement: Figure S4 — Endogenous ANRASSF1 interaction with PRC2. (A) Endogenous levels of ANRASSF1 bound to PRC2 were measured in HeLa cells through RNA IP using an anti-EZH2 antibody, and the results were referred to as % input. The IgG from non-immunized mouse was included as a control. The asterisk indicates non-detectable amounts. (B) The level of lincRNA SFPQ, which does not bind to the PRC2 complex, as determined using anti-SUZ12 and anti-EZH2 antibodies [18], was measured as a positive control. These data show the means ± SD from three independent experiments. (TIF) [file pgen.1003705.s004.tif]

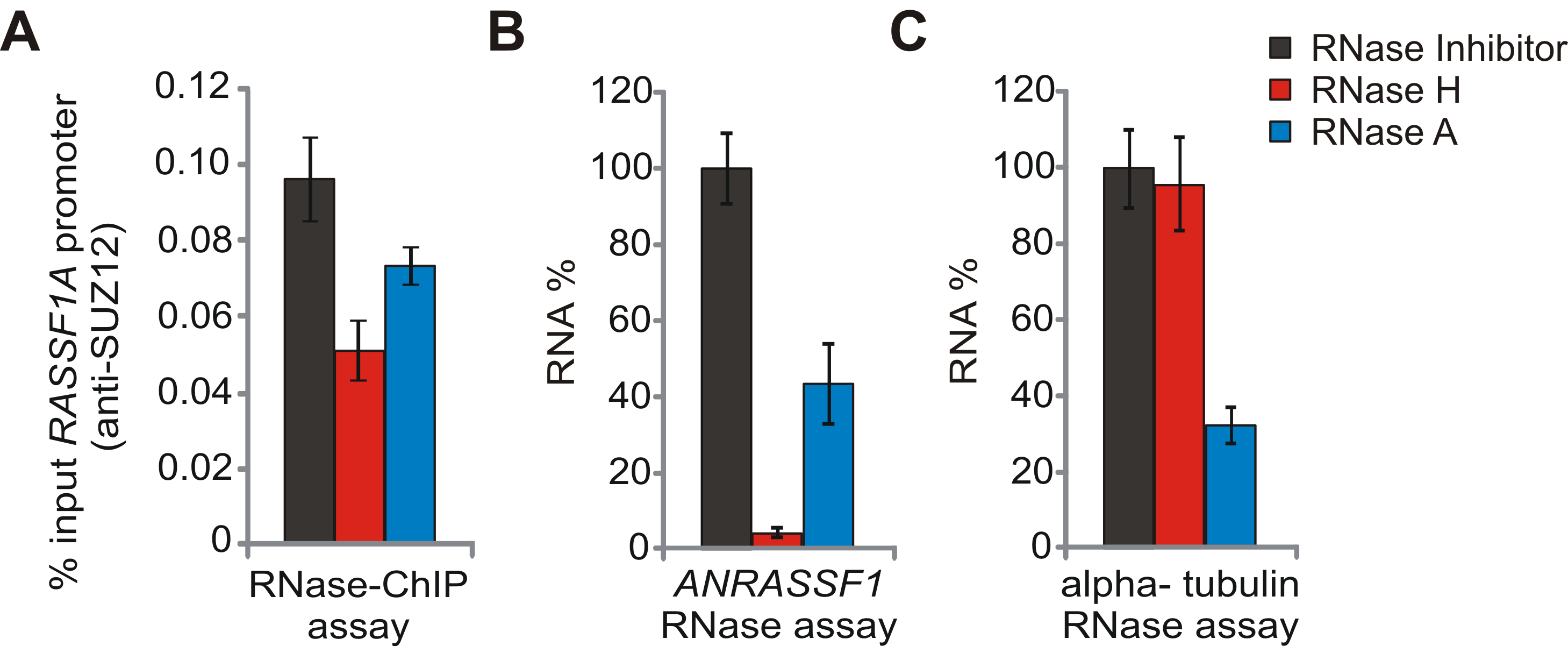

Supplement: Figure S5 — ANRASSF1 mediates the recruitment of SUZ12 to the RASSF1A promoter region in HeLa cells overexpressing ANRASSF1. (A) RNase-ChIP assay for the RASSF1A promoter region measured through qPCR in DNA immunoprecipitated using an anti-SUZ12 antibody in permeabilized HeLa cells overexpressing ANRASSF1. The cells were treated with either RNase inhibitor (black bar), RNase H (red bar) or RNase A (blue bar). The amount of DNA at the RASSF1A promoter region detected through qPCR in anti-SUZ12 samples was calculated in relation to the input. These data show the means ± SD from two independent experiments in triplicate. (B) Detection of ANRASSF1 using RT-qPCR with samples obtained from HeLa cells overexpressing ANRASSF1 and previously permeabilized and treated with RNase inhibitor (black bar), RNase H (red bar) or RNase A (blue bar). The RNA% for each of the two RNase treatments was expressed relative to the corresponding values for RNase inhibitor. These data show the means ± SD from three independent experiments. (C) As a control, alpha-tubulin mRNA was measured in parallel with RT-qPCR under the same conditions as described in (B). These data show the means ± SD from three independent experiments. (TIF) [file pgen.1003705.s005.tif]
