# Supplementary material for: The Intronic Long Noncoding RNA ANRASSF1 Recruits PRC2 to the RASSF1A Promoter, Reducing the Expression of RASSF1A and Increasing Cell Proliferation
Source: PLoS Genet. 2013 Aug 22;9(8):e1003705. doi: 10.1371/journal.pgen.1003705 (PMC3749938; doi:10.1371/journal.pgen.1003705)
Supplement: Protocol S1 — Materials and methods for RACE and primer-walking PCRs, 5′-cap analysis, RNA polymerase transcription inhibition, cell fractionation and the promoter assay. (DOC) [file pgen.1003705.s006.doc]

**Protocol S1. Materials and Methods for race and primer-walking PCR, 5’-cap analysis, RNA polymerase transcription inhibition, cell fractionation and promoter assay.**

Race and primer-walking PCR

For RACE-PCR we used a commercial Human Colon Marathon-Ready cDNA library (Clontech) prepared from poly(A) RNA and Advantage 2 polymerase (Clontech) according to the manufacturer’s instructions. To perform primer-walking PCR and obtain further sequence at the 5´-end, an *in silico* prediction of the transcriptional start site (TSS) was obtained with **TSSW** tool ([http://www.softberry.com](http://www.softberry.com/)), using the sequence of the putative promoter region as an input. We designed a specific forward primer to the predicted TSS, and an alternative forward primer 30 nt upstream to this region. PCR was performed with each of these specific primers in combination with a strand-specific reverse primer that annealed to the known portion of the cDNA near its 5’-end. Only the forward primer at the TSS site gave a product, which we assumed to be the full-length sequence of *ANRASSF1* at the 5’-end. All primers are listed in Table S1.

5’-cap analysis

To test for the presence of a 5’ cap, HeLa total RNA (10µg) was treated with Terminator 5’-phosphate-dependent exonuclease (5’exo, 1 unit; Epicentre Biotechnologies); this is a processive 5´→3´ exonuclease that digests RNA with a free 5´-monophosphate end, and cannot digest RNA that has a 5´-cap. Samples were treated with 5’exo for 2 h at 30°C, according to the manufacturer’s protocol. As a positive control, samples were pretreated with tobacco acid pyrophosphatase (TAP, 10 units; Epicentre Biotechnologies) for 1 h at 37°C to release the 5’ cap, prior to treatment with 5’exo nuclease. Subsequently, reverse transcription was performed with oligo-dT primer plus a specific primer for snRNA U15A using 1µg total RNA with the Super Script III kit protocol (Invitrogen). End-point PCR was performed with specific primers (listed in Table S1). snRNA U15A, which does not have a 5’ cap was used as a negative control for the cap assay.

RNA polymerase transcription inhibition

For RNA Polymerase II inhibition, 5x10^5^ cells seeded per 10-cm-diameter dish were cultured for 24 hrs. Subsequently, the medium was replaced by fresh medium with 10 μg/ml α-amanitin (Sigma) or vehicle (water) and the cells were treated for 24 h. Subsequently, cells were washed once with ice-cold phosphate-buffered saline, harvested, pelleted and total RNA was extracted. RT-qPCR was used for measuring specific genes (primers in Table S1).

For Actinomycin D treatment, 5x10^5^ cells seeded per 10-cm-diameter dish were cultured for 24 h in regular medium; subsequently, it was replaced by fresh medium with 10 μg/ml Actinomycin D (Invitrogen) or vehicle (DMSO). At each time point (0, 1, 3, 6 and 8 h) cells from a control dish and a treated dish were harvested for RNA extraction. RT-qPCR was used for measuring specific genes (primers in Table S1). Half-lives were calculated by fitting a nonlinear regression model of one-phase exponential decay (constraint = plateau >0) using GraphPad Prism software version 5.04.

Cell fractionation

Nuclear and cytoplasmic fractionation and RNA isolation were performed as described . Adequate cell fractionation was controlled by measuring at least 500-fold enrichment of MALAT1 gene ncRNA in the nuclear fraction and, by detecting the 32S rRNA band exclusively in the nuclear total RNA fraction separated by electrophoresis in the BioAnalyzer (Agilent).

Promoter Assay

Putative DNA promoter sequences were amplified (primers in Table S1) from genomic DNA and cloned into pGEM-T vector (Promega); they were excised from the vector and were inserted into pGL3-Basic vector using *Bgl*II and *Hind*III sites. Firefly luciferase assays were performed in 96-well white plates using Dual-Luciferase Reporter Assay System (Promega) according to the manufacturer’s protocol. Firefly luciferase activity was normalized against *Renilla* luciferase, which was expressed from a co-transfected vector, to control for transfection efﬁciency.

**Supporting References**

1. Tycowski KT, Shu MD, Steitz JA (1993) A small nucleolar RNA is processed from an intron of the human gene encoding ribosomal protein S3. Genes Dev 7: 1176-1190.

2. Topisirovic I, Culjkovic B, Cohen N, Perez JM, Skrabanek L, et al. (2003) The proline-rich homeodomain protein, PRH, is a tissue-specific inhibitor of eIF4E-dependent cyclin D1 mRNA transport and growth. EMBO J 22: 689-703.

3. Gondran P, Amiot F, Weil D, Dautry F (1999) Accumulation of mature mRNA in the nuclear fraction of mammalian cells. FEBS Lett 458: 324-328.
