# Supplementary material for: The Intronic Long Noncoding RNA ANRASSF1 Recruits PRC2 to the RASSF1A Promoter, Reducing the Expression of RASSF1A and Increasing Cell Proliferation
Source: PLoS Genet. 2013 Aug 22;9(8):e1003705. doi: 10.1371/journal.pgen.1003705 (PMC3749938; doi:10.1371/journal.pgen.1003705)
Supplement: Table S1 — Primers used for strand-specific RT, cloning, 5′-cap assay, RACE and primer walking. (DOC) [file pgen.1003705.s007.doc]

**Table S1. Primers used for strand-specific RT, cloning, 5’-cap assay, RACE and primer walking**

| **Target** | **Primer name** | **Sequence** |
| --- | --- | --- |
| **Strand specific RT** | | |
| *ANRASSF1*-SENSE | ANRASSF1_Sense_F | CCGCGCAGAATTAGCCTCT |
| *ANRASSF1*-ANTISENSE | ANRASSF1_Antisense_R | CCAATGAGGAAAGGGGAAGT |
| **Cloning (overexpression and promoter assay)** | | |
| Antisense-*ANRASSF1* | ANRASSF_KpnI_F | TGGGTACCGCAGCGGGTGGAGTACTTG |
|  | ANRASSF_HindIII_R | TGAAGCTTCCAATGAGGAAAGGGGAAGT |
| Antisense-Promoter | pANRASSF_F_BglII-AS | AGATCTGACCCAGGACGCGGCAAC |
|  | pANRASSF_R_HindIII-AS | AAGCTTCCAGAGGCCACTCTTGTGC |
| Sense-Promoter | pANRASSF_F_HindIII-S | AAGCTTGACCCAGGACGCGGCAAC |
|  | pANRASSF_R_BglII-S | AGATCTCCAGAGGCCACTCTTGTGC |
| **Cap assay** | | |
| *snRNA U15A* | snRNA U15A_F | GAAGAGATGATGACGAGTCTGACTTG |
| *snRNA U15A* | snRNA U15A_R | GAAATTACTTCAACCAGGGCTCTTT |
| *ANRASSF1* | ANRASSF1_RT_F | GGCAATTAGAACGCTCCTTG |
| *ANRASSF1* | ANRASSF1_RT_R | CTGTGCTAGGCGATAGAGATCC |
| *α-TUBULIN* | α-TUBULIN_RT_F | tcaacaccttcttcagtgaaacg |
| *α-TUBULIN* | α-TUBULIN_RT_R | agtgccagtgcgaacttcatc |
| **RACE and Primer Walking** | | |
|  | ANRASSF_ RACE(3)External_R | ACCTCACACTGCTACGCGGACT |
|  | ANRASSF_RACE(3)_nested_R | GCCTGCTCAACAGTTGGATCTC |
|  | ANRASSF_RACE(5)_External_F | GAGCGTTCTAATTGCCGATTTCC |
|  | ANRASSF_RACE(5)_nested_F | CGGCGGCACAGAGAGGCTAATTCTGCG |
|  | ANRASSF_Walk_F1 | GTCCGCTTGCAGCGGGTG |
|  | ANRASSF_Walk_F2 | AGCCTGGGTCAGCCTGGG |
|  | ANRASSF_Walk_F3 | CACAAGAGTGGCCTCTGGC |
|  | ANRASSF_Walk_F4 | ACAGACCCCACCTACCACAG |
|  | ANRASSF_Walk_F10 | GGAAGGTGCGGGAAGTG |
|  | ANRASSF_Walk_TSS_F1 | GGAATGACCTCATCGCTCCG |
|  | ANRASSF_Walk_TSS_F2 | CAACCGTTAAGACTGAAACGTAGATCG |
|  | ANRASSF_Walk_TSS_F3 | ACGCACGCTTCGCCCC |
|  | ANRASSF_Walk_R | GTATGGGAAGGGCGAGGAT |
