# Supplementary material for: The Intronic Long Noncoding RNA ANRASSF1 Recruits PRC2 to the RASSF1A Promoter, Reducing the Expression of RASSF1A and Increasing Cell Proliferation
Source: PLoS Genet. 2013 Aug 22;9(8):e1003705. doi: 10.1371/journal.pgen.1003705 (PMC3749938; doi:10.1371/journal.pgen.1003705)
Supplement: Table S2 — Primers used for qPCR, ChIP, RIP, RNase-ChIP and methylation assays. (DOC) [file pgen.1003705.s008.doc]

**Table S2. Primers used for qPCR, ChIP, RIP, RNase-ChIP and methylation assays**

| **Target** | **Primer name** | **Sequence** |
| --- | --- | --- |
| **qPCR** | | |
| *ANRASSF1* | ANRASSF1_RT_F | GGCAATTAGAACGCTCCTTG |
|  | ANRASSF1_RT_R | CTGTGCTAGGCGATAGAGATCC |
| *RASSF1C* | RASSF1C_RT_F | GAAATGACCTGGAGCAGCAC |
|  | RASSF1C_RT_R | TTGATCTGGGCATTGTACTCC |
| *RASSF1A* | RASSF1A_RT_F | ACCTCTGTGGCGACTTCATC |
|  | RASSF1A_RT_R | GTTCGTGTCCCGCTCCAC |
| *MALAT1* | MALAT1_RT_F | TTTGATCTAGCACAGACCCTTCAC |
|  | MALAT1_RT_R | GACCTCGACACCATCGTTACCT |
| *LincSFPQ* | LincSFPQ_RT_F | tcgtactgttaggcccttgg |
|  | LincSFPQ_RT_R | aaccttgcatgaagagcacc |
| *HOTAIR* | HOTAIR_RT_F | GAGAGCCAGAGGAGGGAAGAG |
|  | HOTAIR_RT_R | CACGTTTGTTCCGGGAACTG |
| *α-TUBULIN* | α-TUBULIN_RT_F | tcaacaccttcttcagtgaaacg |
|  | α-TUBULIN_RT_R | agtgccagtgcgaacttcatc |
| *pre-tRNA^Tyr^* | pre-tRNA^Tyr^_F | AAAAAACCGCACTTGTCTCCTTCG |
|  | pre-tRNA^Tyr^_R | CCTTCGATAGCTCAGCTGGTAGAG |
| *c-Myc* | c-Myc_RT_F | TCAAGAGGTGCCACGTCTCC |
|  | c-Myc_RT_R | TCTTGGCAGCAGGATAGTCCTT |
| HPRT1 | HPRT1_F | TGACACTGGCAAAACAATGCA |
|  | HPRT1_R | GGTCCTTTTCACCAGCAAGCT |
| **ChIP** | | |
| Promoter *HYAL2* | Promoter- HYAL2_F | GACGTGGCCGCCCATT |
|  | Promoter- HYAL2_R | CTCGTGGCTGAGACTCCCATA |
| Promoter *TUSC2* | Promoter- TUSC2_F | GGCTGTCTCCACGGAAACC |
|  | Promoter- TUSC2_R | CCCCACGACTTGAGCAACA |
| Promoter *RASSF1C* | Promoter-RASSF1C_F | TTCGCGCGGTGAAGTACTG |
|  | Promoter-RASSF1C_R | GCGCCTTCTTTCGAAATGAC |
| Promoter *RASSF1A* | Promoter-RASSF1A_F | TAGAGGAAGAGGGTCCCCACAT |
|  | Promoter-RASSF1A_R | TGGGTAGGCCAAGTGTGTTG |
| Promoter *ZMYND10* | Promoter- ZYMD10_F | AATCCGGGCGGGTCTCTAG |
|  | Promoter- ZYMD10_R | GAAAGCGCTGGGAATCCAA |
| Promoter *NPRL2* | Promoter- NPRL2_F | GCCTCACAGTTGTCTGCGAAT |
|  | Promoter- NPRL2_R | GCTCAACTGGCTGCCTGAA |
| Promoter *HOXA9* | Promoter- HOXA9_F | TGACCTTGAATGGCCCAAAG |
|  | Promoter- HOXA9_R | CTGGTCACAGCAGGTAGGGTTAG |
| Promoter *GAPDH* | Promoter- GAPDH_F | TACTAGCGGTTT TACGGGCG |
|  | Promoter- GAPDH _R | TCGAACAGGAGGAGCAGAGAGCGA |
| **Methylation** | | |
| Promoter *RASSF1A* | Promoter-RASSF1A_F | TAGAGGAAGAGGGTCCCCACAT |
|  | Promoter-RASSF1A_R | TGGGTAGGCCAAGTGTGTTG |
