# Supplementary material for: The Intronic Long Noncoding RNA ANRASSF1 Recruits PRC2 to the RASSF1A Promoter, Reducing the Expression of RASSF1A and Increasing Cell Proliferation
Source: PLoS Genet. 2013 Aug 22;9(8):e1003705. doi: 10.1371/journal.pgen.1003705 (PMC3749938; doi:10.1371/journal.pgen.1003705)
Supplement: Table S3 — Oligonucleotide sequences used for knockdown assays. (DOC) [file pgen.1003705.s009.doc]

**TableS3. Oligonucleotide sequences used for knockdown assays**

| **Target** | **Oligo name** | **Sequence** |
| --- | --- | --- |
| *ANRASSF1* | stealth_ANRASSF_1 | GGGAAAUCGGCAAUUAGAACGCUCC |
| *ANRASSF1* | stealth_ANRASSF_1 | GGAGCGUUCUAAUUGCCGAUUUCCC |
|  | stealth_scrambled_1 | GGGCUACGGUAAGAUCAACGAAUCC |
|  | stealth_scrambled_1 | GGAUUCGUUGAUCUUACCGUAGCCC |
| *ANRASSF1* | stealth_ANRASSF_3 | UGGAUCUCUAUCGCCUAGCACAGAA |
| *ANRASSF1* | stealth_ANRASSF_3 | UUCUGUGCUAGGCGAUAGAGAUCCA |
|  | stealth_scrambled_3 | UGGUCUCGCUAAUCCCACGAUAGAA |
|  | stealth_scrambled_3 | UUCUAUCGUGGGAUUAGCGAGACCA |
| *ANRASSF1* | stealth_ANRASSF_2 | CGACCUAUCUCAGUGGGUUACCUCA |
| *ANRASSF1* | stealth_ANRASSF_2 | UGAGGUAACCCACUGAGAUAGGUCG |
|  | stealth_scrambled_2 | CGAAUCUCUGAGUGGUUCACCCUCA |
|  | stealth_scrambled_2 | UGAGGGUGAACCACUCAGAGAUUCG |
| *ANRASSF1* | ANRASSF1_ASO_3^§^ | mU*mC*mU*mG*mU*G*C*T*A*G*G*C*G*A*T*mA*mG*mA*mG*mA |
|  | scrambled_ASO^§^ | mA*mC*mU*mA*mC*C*G*A*U*C*C*A*C*A*C*mU*mC*mU*mU*mU |

**^§^ (**m) corresponds to 2'-O-Methyl nucleotide and (*) to phosphorothioate backbone modifications.
